# A Conserved Multi-Gene Family Induces Cross-Reactive Antibodies Effective in Defense against *Plasmodium falciparum*

**DOI:** 10.1371/journal.pone.0005410

**Published:** 2009-04-30

**Authors:** Subhash Singh, Soe Soe, Simon Weisman, John W. Barnwell, Jean Louis Pérignon, Pierre Druilhe

**Affiliations:** 1 Bio-medical Parasitology Unit, Institut Pasteur, Paris, France; 2 Malaria Branch, Division of Parasitic Diseases, Centers for Disease Control and Prevention, Atlanta, Georgia, United States of America; Initiative for Vaccine Research, Switzerland

## Abstract

**Background:**

Two related merozoite surface proteins, MSP3 and MSP6, have previously been identified as targets of antibody-dependent cellular inhibition (ADCI), a protective mechanism against *Plasmodium falciparum* malaria. Both MSP3 and MSP6 share a common characteristic small N-terminal signature amino-acid stretch (NLRNA/G), a feature similar to MSP3-like orthologs identified in other human and primate malaria parasites.

**Methods/Results:**

This signature amino-acid sequence led to the identification of eight ORFs contiguously located on *P. falciparum* chromosome 10. Our subsequent investigations on their expression, localization, sequence conservation, epitope sharing, immunogenicity and the functional role of antibodies in defense are reported here. Six members of *P. falciparum* MSP3-multigene family share similar sequence organization within their C-terminal regions, are simultaneously expressed as merozoite surface proteins and are highly conserved among parasite isolates. Each of these proteins is a target of naturally occurring antibodies effective at parasite killing in ADCI assays. Moreover, both naturally occurring antibodies and those generated by immunization display cross-reactivity with other members of the family and exhibit varied binding avidities.

**Conclusions/Significance:**

The unusual characteristics of the MSP3 multi-gene family lead us to hypothesize that the simultaneous expression of targets eliciting cross-reactive antibody responses capable of controlling parasite densities could represent an immune process selected through evolution to maintain homeostasis between *P. falciparum* and human hosts; a process that allows the continuous transmission of the parasite without killing the host. Our observations also have practical consequences for vaccine development by suggesting MSP3 vaccine efficacy might be improved when combined with the various C-terminus regions of the MSP3 family members to generate a wider range of antibodies acting and to increase vaccine immunogenicity in varied human genetic backgrounds.

## Introduction

Naturally acquired immunity against the asexual blood stages of human malaria is well-documented [1]. It is a state of immunity against malaria, whereby immune effector mechanisms maintain low densities of parasites and conversely chronic infection appears necessary for long-term maintenance of effective clinical protection. Hence it has been named “premunition” in order to reflect this incomplete effect and a delicately balanced equilibrium [2]. Passive transfers of immune sera to patients have demonstrated that premunition is mediated by antibodies as IgG from clinically immune individuals can control parasite density and disease symptoms in recipients [3]–[6]. The same effect was obtained against parasites from different geographical settings suggesting conservation of the antigenic targets in different parasite strains.

A careful study of immune effectors against *P. falciparum*, and of the protective versus non-protective antibodies in one such passive transfer experiment, led us to characterize the Antibody Dependent Cellular Inhibition of parasite multiplication (ADCI) as a major mechanism underlying premunition [7]. Indeed we found that IgG from hyperimmune individuals inhibited *P. falciparum* growth *in vitro* in co-operation with blood monocytes [8]. This ADCI effect, validated by a clinical experiment, thereafter served as a functional assay for the identification of the targets of protective IgG. A novel antigen, Merozoite Surface Protein 3 (MSP3) was identified in this manner by screening a *P. falciparum* genome-wide expression library using ADCI [9]. In contrast to nearly all other malaria vaccine candidates, the MSP3 C-terminal half showed complete sequence conservation among >100 *P.falciparum* field isolates from different geographical regions [9].

The choice of MSP3 as a malaria vaccine candidate has been further supported by results from a series of immuno-clinical field studies, immunization of pre-clinical models and of volunteers in clinical trials, with assessment of functional anti-parasite activity. Seven studies in several field settings from Asia and Africa have shown that anti-MSP3 antibodies, particularly belonging to the IgG3 subclass, were strongly associated with acquired clinical protection against malaria [10]–[14]. A human recombinant anti-MSP3 antibody was found able to achieve parasite killing in co-operation with monocytes at nanomolar concentrations [15]. Protection has been induced by MSP3 in primates challenged by *P.falciparum* [16]. A phase-I MSP3 vaccine trial in 36 volunteers elicited antibodies in humans that mediated efficient killing of *P. falciparum* both *in vitro* and *in vivo* [17], and further Phase Ib studies in African populations have confirmed the safety and immunogenicity of the formulation [18], [19].

Subsequently, another Merozoite Surface Protein, called MSP6, was identified with significant sequence similarities with MSP3 within its conserved C-terminal region. Antigenic domains within the related C-terminal regions of MSP3 and MSP6 either shared near complete sequence identity and cross-reactivity or limited diversity with distinct antigenic properties both being targets of naturally occurring antibodies capable of mediating parasite killing [20]. Moreover, the C-terminal region of MSP6 is also highly conserved in different parasite isolates.

Both MSP3 and MSP6 sequences show a small stretch of amino-acids (NLRNA/G) immediately following their predicted N-terminal signal sequence, a feature shared by MSP3 orthologs identified from *P. vivax* and *P. knowlesi*, [21], [22]. We therefore used this small stretch of amino acids as ‘signature sequence’ in the N-terminal region as a selection criterion and identified a series of open reading frames located on chromosome 10 sharing an organization of their C-terminal regions similar to that of MSP3.

In view of the vaccine potential of MSP3, particularly the encouraging results obtained in the clinical vaccine trials, we decided to investigate in detail the new members of this family with respect to their expression, localization, sequence conservation, epitope sharing, immunogenicity and functional role of antibodies in defense. Results shows that the MSP3-gene family differs from other *P. falciparum* multi-gene families described so far and suggest that the corresponding proteins play an important role in eliciting protective antibody responses in humans. Based on these results we formulate the hypothesis that the *P. falciparum* MSP3-family of proteins could have the role of ensuring both host and parasite survival leading to chronic infection without disease symptoms.

## Materials and Methods

### Sequence analysis

The *P. falciparum* 3D7 genome database was searched using GenBank BLAST at NCBI (http://www.ncbi.nlm.nih.gov/Malaria/plasmodiumbl.html). All BLAST searches were done without the low-complexity filter and with all other settings kept at default. Pairwise homology was performed between different protein sequences using Wilbur-Lipman algorithm, PAM 250 using the Gene Jockey II sequence analysis software. ClustalW was used to produce the multiple alignments (http://www.ebi.ac.uk/cgi-bin/newclustalwpl), which were copied into Boxshade Hofmann, Barron (at http://bioweb.pasteur.fr/seqanal.interfaces/boxshade.html#letters) to produce the alignments. Prediction of the signal peptides was done using iPsort and Signal P (at http://hypothesiscreator.net/iPSORT/predict.cgi and http://www.cbs.dtu.dk/services/signalp/#submission, respectively). Prediction and analysis of coiled-coil regions from amino acid sequences was performed with the COILS2.1 program [23]. Predictions of two and three-stranded coiled-coil regions were performed with the PAIRCOIL based MULTICOIL program [24]. Leucine zipper predictions were based on the LZpred program [25] that combines a coiled-coil prediction algorithm with an approximate search for the characteristic leucine repeat. Regions of least sequence relatedness among different members of the MSP3-family of proteins were identified as ‘unique regions’ consisting of 50–80 residues (SI, Table 1A). The ‘unique regions’ did not show significant relatedness to the amino-acid sequence of any other *P. falciparum* protein in the database as analyzed through BLASTP searches (‘score bit’ value to themselves in the BLAST was always greater than 100 with ‘E values’ in the range of 9e-23 to 1e-40; a few other hits obtained only against some of the *ORF*s denominated *MSP3.3*, *MSP3.4* and *MSP3.5* were with a low ‘score bit’ value of less than 40 with ‘E values’ not less than 1e-04).

**Table 1:** BLASTP comparison of the *P. falciparum* MSP3 family of proteins.

Gene product	E value (% identity, % similarity)					
	MSP3.1	MSP3.2	MSP3.3	MSP3.4	MSP3.7	MSP3.8
MSP3.1	0.0	9×10^−41^ (31,48)	3×10^−21^ (26,42)	3×10^−21^ (26,48)	8×10^−24^ (25,40)	2×10^−17^ (25,41)
MSP3.2	8×10^−41^ (31,48)	0.0	1×10^−21^ (30,47)	2×10^−23^ (27,45)	7×10^−29^ (26,43)	9×10^−23^ (31,51)
MSP3.3	9×10^−27^ (26,42)	9×10^−22^ (30,47)	0.0	1×10^−17^ (32,51)	2×10^−20^ (25,41)	1×10^−17^ (27,45)
MSP3.4	1×10^−21^ (26,48)	1×10^−24^ (27,45)	5×10^−18^ (32,51)	0.0	2×10^−17^ (31,52)	1×10^−92^ (31,47)
MSP3.7	7×10^−24^ (25,40)	3×10^−29^ (26,43)	-	3×10^−17^ (31,52)	0.0	9×10^−12^ (25,40)
MSP3.8	7×10^−19^ (25,41)	2×10^−23^ (31,51)	1×10^−18^ (27,45)	6×10^−93^ (31,47)	2×10^−12^ (25,40)	0.0

The sequences indicated in bold were used as queries in a custom blast at the Malaria Genetics/Genomic database at NCBI.

### *MSP3*-family gene polymorphism analysis

Genomic DNA was isolated from 36 *P. falciparum* field isolates obtained from different parts of the world: Brazil (B); Comoro Islands (C); Dielmo, Senegal, West Africa (D) and Thailand (T), using standard procedure, or commercially available kit (Qiagen). *MSP3-family* gene fragments were PCR amplified from these DNA samples by using the primer pairs (SI, Table 1B). A control (without template DNA) was included for each round of PCR to ensure absence of PCR contamination. PCR amplicons were purified by agarose gel electrophoresis, and sequenced using standard dye-termination sequencing techniques. ClustalW alignment of the predicted amino-acid sequence for each member of the *MSP3-family* among different isolates is shown (SI, Sequence conservation).

### Cloning and expression of recombinant proteins

‘Unique region’ and ‘related carboxy-terminal’ gene fragments were cloned from 3D7 strain genomic DNA and expressed and purified as N-terminal His-tagged recombinant proteins. A set of eight His-tagged recombinant proteins covering the unique regions from each of the eight ORFs (MSP3.1u, MSP3.2u, MSP3.3u, MSP3.4u, MSP3.5u, MSP3.6u, MSP3.7u and MSP3.8u) were designed and expressed (Table S1). A set of six recombinant proteins were designed to cover the related C-terminal regions of the six genes showing substantial homology within their C-terminal regions, and named MSP3.1-Ct, MSP3.2-Ct, MSP3.3-Ct, MSP3.4-Ct, MSP3.7-Ct and MSP3.8-Ct (Table S2).

### RNA analysis

RNA was extracted from asynchronous blood stage parasite culture (3D7, harvested at 10–15% parasitemia) using TRIZOL (Invitrogen), according to the manufacturer's instructions. RNA pellets were stored at −70°C. To ensure that RNA was completely free of contaminating DNA, it was treated with DNaseI using DNA-free kit (Ambion). First-strand of cDNA was synthesized from around 1 µg of DNA-free RNA using a set of random primers and M-MLV Reverse transcriptase (Invitrogen) following the supplier's instructions. Amplification of the unique regions of MSP3-family of genes was done using the set of primers listed in Table S1. Controls consisting of genomic DNA as template and no nucleic acid (water as template) were included for each primer pair.

### Western blot and dot-blot assays

Western blot analysis was performed against parasite proteins resolved on a 12% SDS-PAGE under denaturing conditions using standard protocols as described elsewhere [26]. Dot-blot assays were performed using purified recombinant proteins on strips of nitrocellulose paper (Amersham). In order to obtain comparable protein distribution of proteins in each dot sample, both the concentration and volume was adjusted. Typically 2 µg of purified recombinant protein in 10 µl was applied to the nitrocellulose membrane using a vacuum manifold (BioRad). Dot-blots were processed for antibody signal detection similar to the Western blot strips.

### Indirect Immunofluorescence Assay (IFA)

IFA was performed on air-dried, acetone-fixed, thin smears of *P. falciparum* mature schizonts. IFA was used to detect subcellular localization of proteins and to adjust the functional concentration of the affinity-purified antibodies for use in ADCI assays, as described elsewhere [12].

### ELISA

ELISA assays were performed for the detection of total IgG and subclasses, as described elsewhere [27] in a pool of hyperimmune sera against different members of the MSP3-family of proteins. Sera from mice immunized with MSP3.1-Ct and MSP3.2-Ct recombinant proteins were also tested for their ability to cross-react with other members of the MSP3-family. The specific reactivity of each serum sample (human/mouse) was obtained by subtracting the optical density value of a control protein (0.25 µg of bovine serum albumin/well) from that of the test antigens.

### Affinity purification of antibodies

Antibodies were affinity-purified against different members of the MSP3-family of proteins from a pool of hyperimmune sera obtained from the inhabitants of the village of Dielmo, Senegal, West Africa. Affinity purification was done as described elsewhere [12] using purified recombinant protein adsorbed on the surface of polystyrene beads (mean diameter, 10 µm; Polysciences). Specific antibodies were eluted by use of 0.2 M glycine (pH 2.5) and were immediately neutralized to pH 7.0 using 2 M aqueous Tris-base. Affinity-purified antibodies were dialyzed extensively against PBS followed by RPMI and were concentrated using Centricon concentrators (Millipore), filter sterilized, and, after addition of 1% Albumax (Invitrogen), stored at 4°C.

### Cross-reactivity studies

The degree of antigenic relatedness between the different carboxy-terminal recombinant proteins from members of the MSP3 family of proteins was assessed by testing the cross-reactivity of antibodies generated against each recombinant protein. To test the existence of antigenic relatedness among different members of the MSP3 family of proteins, ELISA assays were performed using antibodies affinity-purified from hyperimmune sera at a concentration of 10 µg/ml. Cross-reactivity of mice sera generated against MSP3.1-Ct and MSP3.2-Ct recombinant proteins were tested against other members of the MSP3-family of proteins by performing ELISA, using 1∶50 dilution of the mice sera.

### Avidity studies

Antibody binding avidity was determined for naturally occurring human antibodies against the C-terminal recombinant proteins from MSP3-family members using dot-blot assay. Identical strips of nitrocellulose membranes arrayed with equal amount of recombinant proteins were tested for residual antibody binding after no treatment or treatment with increasing concentrations of chaotropic salt (NH_4_SCN: 0.1 M, 0.25 M, 0.62 M, 1.56 M and 3.9 M) for 20 min at room temperature. Values obtained for the antibody reactivity against any antigen in presence of no NH_4_SCN salts was considered to be 100%, and the residual antibody reactivity after treatment with higher concentrations of NH_4_SCN salts was expressed as fractions of this 100%. Quantitative assessment of antibody reactivity was done by Adobe Photoshop based image analysis after scanning the dot-blots using EPSON scanner (model: EU34; EPSON TWAIN software). The image was analyzed with Adobe Photoshop software (version 6, Adobe Systems) using a Macintosh PowerPC G4 system. Briefly, a fixed pixel area was selected from the nitrocellulose membrane containing both the “dot-staining” due to the antibody reactivity together with a portion of “background” (surrounding unstained nitrocellulose membrane). This pixel-area was saved using the “save selection” option (Select-menu) and was used to generate histograms (Image-menu). The histograms were set to display statistical details of the selected pixel area in the “luminosity” channel. The “Std Dev” value in the histogram represented the level of contrast between the bright areas (nitrocellulose background) and the dark area (staining due to antibody reaction), and was used for comparing the levels of residual antibody reactivity.

### Functional *in vitro* antibody assays

The antibody-dependent, monocyte-mediated ADCI assays were performed in duplicate by use of laboratory-maintained strains 3D7 and Uganda Palo-Alto, as described elsewhere [28]. Briefly, monocytes from healthy, non–malaria-exposed donors were prepared as described elsewhere [28]. The affinity-purified antibodies, adjusted to a concentration yielding a 1/200 IFA end-point titer, were added at a rate of 10 µL in 90 µL of complete culture medium, which yielded a final titer of 1/20 in the ADCI assay. After cultivation for 96 h, the level of parasitemia was determined on Giemsa-stained thin smears from each well by the microscopic examination of 50,000 erythrocytes. Monocyte-dependent parasite inhibition is expressed as the specific growth inhibition index (SGI): SGI = 1-([percentage of parasitemia with monocytes and test IgG/percentage of parasitemia with test IgG)/(percentage of parasitemia with monocytes and normal IgG/percentage of parasitemia with normal IgG]). A positive control IgG, from the pool of serum samples from Ivory Coast used for passive-transfer experiments in humans [6] and a negative control IgG, from French donors who were never exposed to malaria infection, were included in the assay.

## Results

### Identification of six MSP3 paralogs located in tandem on Chromosome 10

**Figure 1:**
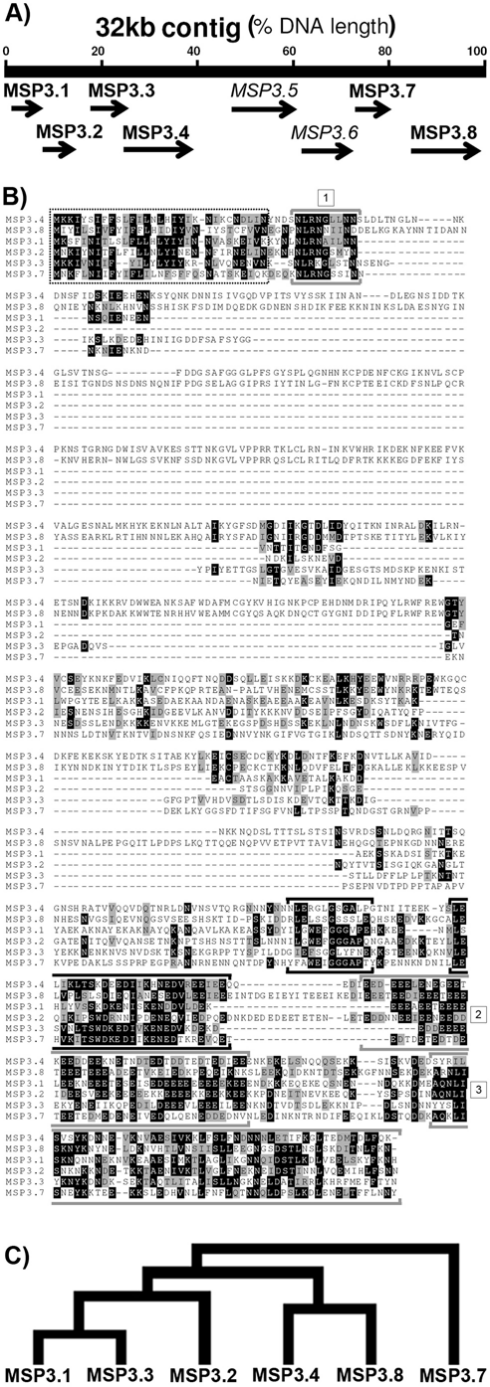
*Panel* (A): Schematic presentation of a 32 kb contig located on *P. falciparum* Chromosome 10 (1404403 to 1436403 bp of the 3D7 strain) indicating the relative positions of the MSP3-like ORFs. MSP3.1 and MSP3.2 are genes known to encode merozoite surface proteins MSP3 and MSP6, respectively. *Panel* (B): ClustalW alignment and Boxshade representation of the amino acid sequences of the MSP3-family of proteins with related C-terminal sequence organization. MSP3.5 and MSP3.6 do not share the C-terminal sequence similarities with other members. Identical residues are indicated by white letters on black backgrounds, whereas similar residues are indicated by black letters on a gray background. Dashes represent gaps to optimize alignments. The pattern shared by MSP3-family members are: the N-terminal signal-peptide (dotted line-box); the signature sequence of the MSP3 family of proteins [1]; the glutamic acid rich region [2]; and the leucine-zipper domain [3]. Sequences highlighted in black are related to regions identified as targets of protective antibodies identified in MSP3.1. *Panel* (C): A cladogram showing sequence analogy between different MSP3-like ORFs derived by comparing the encoded protein sequences.

Based on the signature motif (NLRNA/NLRNG) in the N-terminal region of MSP3, MSP6, and of multi-allelic orthologs of MSP3 from other species [21], our search for MSP3 paralogs in *P. falciparum* genome (http://www.ncbi.nlm.nih.gov/Malaria/plasmodiumbl.html) identified a contig of 32 kb on chromosome 10, with 8 ORFs located contiguously on the same coding strand (Fig. 1A). The PlasmoDB (http://www.plasmodb.org/plasmo/home.jsp) gene IDs for these ORFs are: *PF10_0345 (MSP3)*, *PF10_0346 (MSP6)*, *PF10_0347 (H101)*, *PF10_0348* (hypothetical protein), *PF10_0350* (hypothetical protein), *PF10_0351* (hypothetical protein), *PF10_0352 (H103)* and *PF10_0355* (hypothetical protein), with known genes *GLURP (PF10_0344)* and *LSA-1 (PF10_0356)* flanking on the 5′ and 3′ sides of this cluster, respectively. All of these ORFs have an N-terminal signal peptide region predicted by iPSORT and SignalP-3.0 programs. The contiguous location of these eight ORFs on the same chromosome is suggestive of gene duplication events with subsequent divergences as a possible mechanism for evolution of this gene family.

**Figure 2:**
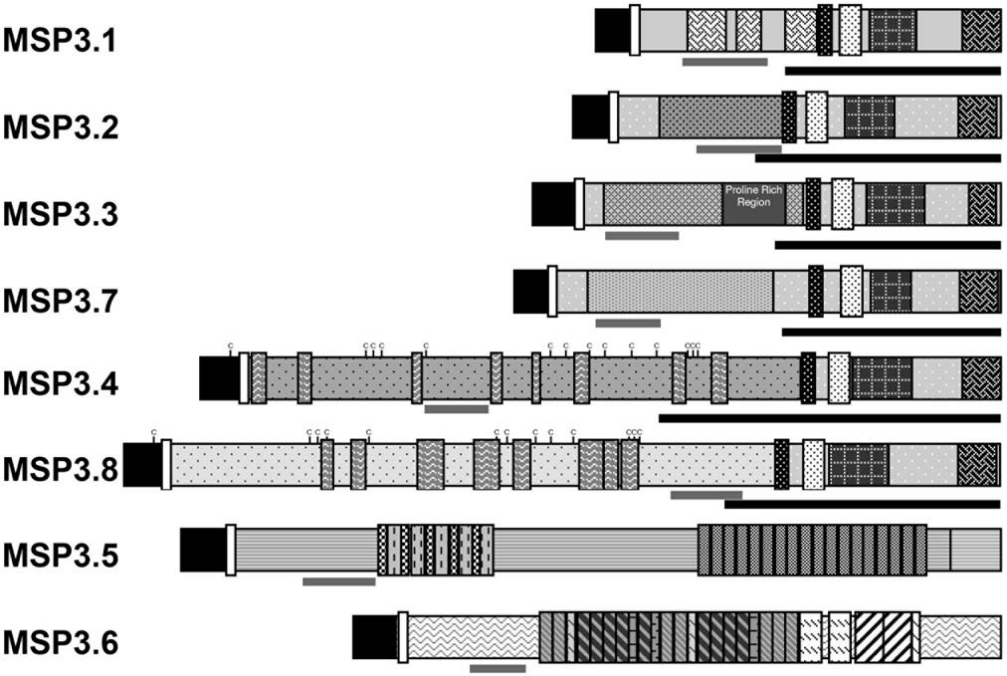
Schematic presentation of the protein sequences encoded by MSP3-like ORFs.

All of these eight ORFs retain the N-terminal MSP3-signature motif, while six of these also display striking sequence relatedness in their C-terminal regions (≈32% identity and ≈54% similarity of amino acid residues, Fig. 1, Fig. 2 and Table 1), marked by a glycine-rich motif and followed by an acidic region and a coiled-coil region, together with domains which have been identified as targets of protective antibodies in MSP3 [12] and MSP6 [20], as shown in Fig. 1B. Based on their sequence relatedness we propose to group these *ORF*s as MSP3-multigene family in *P. falciparum*, and rename them in the order of their location on the chromosome with previously described members: *MSP3* becoming *MSP3.1*, *MSP6* renamed *MSP3.2*, H101 renamed MSP3.3, H103 renamed MSP3.7 and the remaining being *MSP3.4*, *MSP3.5*, *MSP3.6*, and *MSP3.8*. Of these eight ORFs, two of them, *MSP3.5* and *MSP3.6*, shall be distinguished as they do not share relatedness to other members of the family in their C-terminal regions (Fig. 2), and are therefore described separately in the following analysis.

In contrast to the similarities observed within the C-terminus regions of the six main members of the family, the N-terminal regions of these ORFs show high level of sequence divergence from one gene to the other. Two of these, *MSP3.4* and *MSP3.8*, include a DBL-like cysteine-rich domain, similar to those observed in members of the *var* or *ebl* gene families (Fig. 2), which suggests a possible role in interaction with erythrocytes. The characteristic *MSP3.1* heptad repeats and *MSP3.7* proline-rich region also suggest protein-protein interaction roles for these domains.

### High degree of sequence conservation of the C-terminal regions of the 6 genes

As the C-terminal region of MSP3 is characterized by an unusually high degree of sequence conservation, this feature was investigated in the remaining five ORFs. Sequence analysis of the related C-terminal regions in thirty-six *P. falciparum* isolates revealed a remarkably high degree of intra-species amino acid sequence conservation (>95%) among parasite isolates of various geographical origins (Text S1). This high degree of sequence conservation is quite unusual among *P. falciparum* genes coding for surface antigens and, therefore suggests an unknown but strong functional importance of these domains in the parasite biology.

### The six MSP3-like ORFs are expressed as merozoite surface proteins

We analyzed the expression of the *MSP3*-multigene family in *P. falciparum* 3D7 strain, both at RNA and protein level. To this end, “unique regions” (70–80 amino acids stretches chosen as having the least relatedness with the remaining members of the MSP3-family and with other *P. falciparum* proteins, as verified by BLASTP-analysis) were identified within each *ORF*, as shown in Fig. 2, and used to analyze transcription and expression.

PCR analysis of cDNA prepared from asynchronous asexual parasite culture using unique region primer sets (Table S1) showed RNA expression for all six MSP3-family members (Fig. 4, panel A).

**Figure 3:**
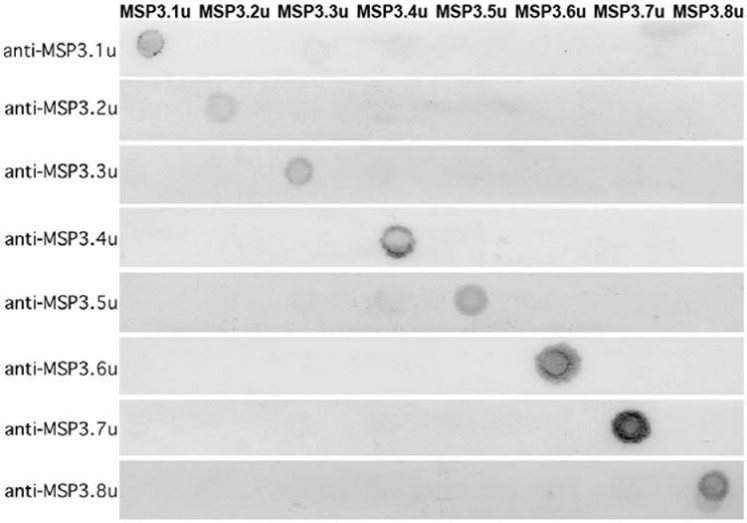
Specificity of antibodies affinity-purified against recombinant proteins covering the ‘unique-regions’ identified in each member of the MSP3-like ORFs. 2 µg of the purified His-tag recombinant protein (MSP3.1u,….MSP3.8u) was dot blotted on nitrocellulose strips. Antibodies affinity-purified against each unique region recombinant protein, from hyperimmune sera (anti-MSP3.1u,….anti-MSP3.8u), were tested against a panel of all unique region recombinant proteins, as shown in the figure above. The pattern of antibody reactivity shows high specificity of the affinity-purified antibodies towards the recombinant proteins against which they were affinity-purified.

**Figure 4:**
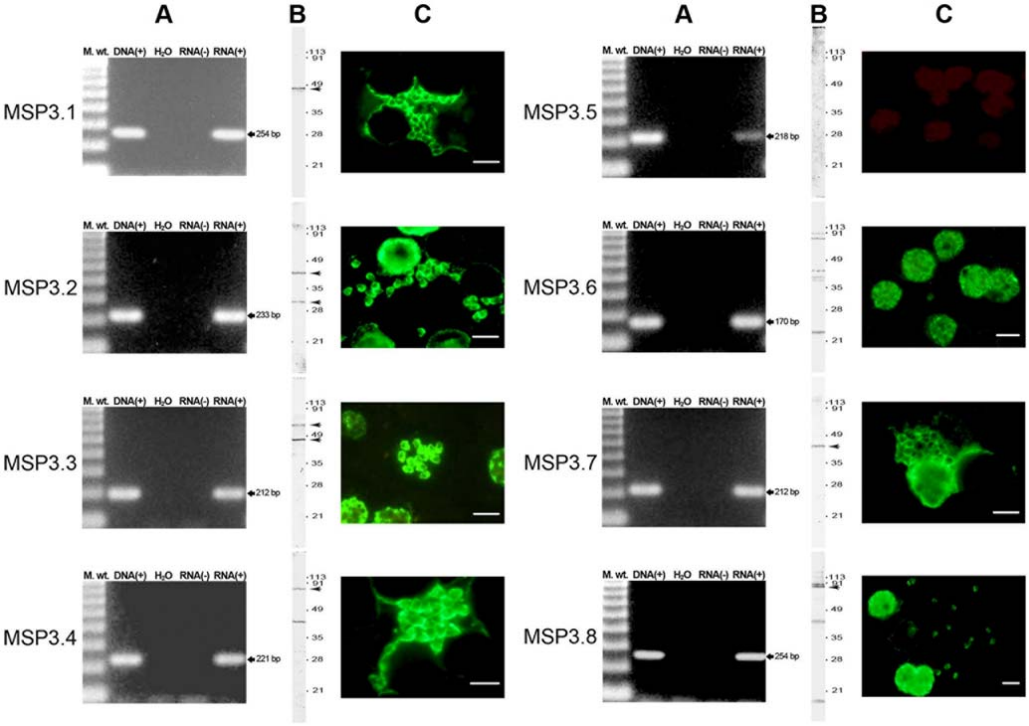
Expression analysis of MSP3-like ORFs. *Panel* A: Transcript analysis by RT-PCR. Arrowheads indicate the size of the cDNA amplification obtained using primer sets specific for each ORF. Note that the transcript for MSP3.5 was less abundant as compared to other members of the family. *Panel* B: Detection of protein encoded by different MSP3-like ORFs in *P. falciparum* 3D7 blood stage extract, by Western blot analysis, using antibodies affinity-purified against unique non cross-reactive region identified in each ORF. Arrowheads indicate the size of the *P. falciparum* protein detected by denaturing SDS-PAGE. The numbers represent positions of the molecular weight markers. Antibodies affinity-purified against unique regions of MSP3.5 and MSP3.6 did not detect specific protein products in the parasite extract. *Panel* C: IFA analysis of acetone-fixed thin smear of the blood stage parasites, using the same antibodies used for Western blot analysis, shows merozoite surface staining. The size-bar drawn in the lower right-hand corner of each microscopic field represents 5 µm. Antibodies affinity-purified against the unique region of MSP3.5 did not react to parasite proteins in IFA.

To test protein expression, these unique regions were also expressed as His-tagged recombinant proteins in *E. coli* (Fig. 2, and Table S1). Then antibodies from African hyperimmune sera were affinity-purified against the unique region recombinant proteins, as described elsewhere [12]. The specificity of antibodies affinity-purified against each unique region recombinant was established by testing its reactivity against the unique regions from the remaining MSP3-family members, and against other blood stage proteins expressed in *P. falciparum* using protein blots (Fig. 3). Western blot analysis using asynchronous asexual blood stage parasite protein was performed using antibodies affinity-purified against each unique region recombinant protein (Fig. 4, panel B). The expression of the native parasite protein was confirmed for the six *ORF*s with related C-terminal region. Though the observed molecular weights of the parasite proteins were mostly in agreement with their calculated molecular weights, in few cases like in MSP3.1 the observed molecular weight (≈48 kDa) was higher than the calculated molecular weight (≈40 kDa), which is in keeping with previous observations [29]. Lower molecular weight proteins, recognized by some affinity-purified antibodies, could be due to the proteolytic processing of the nascent protein as known in the case of MSP3.2 [30], or degradation products.

Previous studies have demonstrated the merozoite surface localization for MSP3.1 and MSP3.2 [9], [20]. Using antibodies affinity-purified against the unique regions, we assessed the localization of other members of the family in maturing schizonts and released merozoites of *P. falciparum* 3D7 strain, through IFA (Fig. 4, panel C). Antibodies against all the six ORFs with related C-terminal region stained the surface of free merozoites, showing a pattern indistinguishable from that observed for MSP3.1 or MSP3.2. This pattern of IFA reactivity was further confirmed in two other laboratory strains, Uganda Palo-Alto and T23 (data not shown). Each of the parasites examined under several microscopic fields showed fluorescent labeling when stained by antibodies against unique regions of different members, indicating consistent expression of the six proteins in all individual parasites.

Among the two ORFs, which did not share the C-terminal relatedness, *i.e.* MSP3.5 and MSP3.6, RNA expression was also detected. However, the low level of cDNA amplification observed for MSP3.5 suggests lower stability of the corresponding transcript (Fig. 4, panel A, top right). Furthermore, antibodies affinity-purified against MSP3.5u and MSP3.6u recombinant proteins did not recognize specific parasite proteins. Whereas anti-MSP3.5 antibodies did not react to any parasite protein, anti-MSP3.6u antibodies reacted to several polypeptides, which did not match its calculated molecular weight of 65 kDa (Fig. 4, panel B). Anti-MSP3.5u antibodies also failed to react to the parasite protein by IFA (Fig. 4, panel C, top right). It is likely that owing to its less stable transcript, MSP3.5 is not expressed in the erythrocytic stage. Anti-MSP3.6u antibodies reacted to mature schizonts and free merozoites (Fig. 4, panel C). However, since anti-MSP3.6u antibodies displayed obvious cross-reactivities to several parasite proteins in Western blot analysis, its expression remains to be investigated in greater detail. These results confirm that these two ORFs differ markedly from the other six.

Conversely, results from RNA and protein expression analysis, together with cellular localization studies demonstrate that the six MSP3-family members with C-terminal relatedness are all expressed in the erythrocytic stage of parasite development as merozoite surface proteins, thus, constituting the MSP3-family of proteins in *P. falciparum* asexual blood stage development.

### A network of cross-reactivity among MSP3-family members is displayed by naturally occurring or immunization-induced antibodies

**Figure 5:**
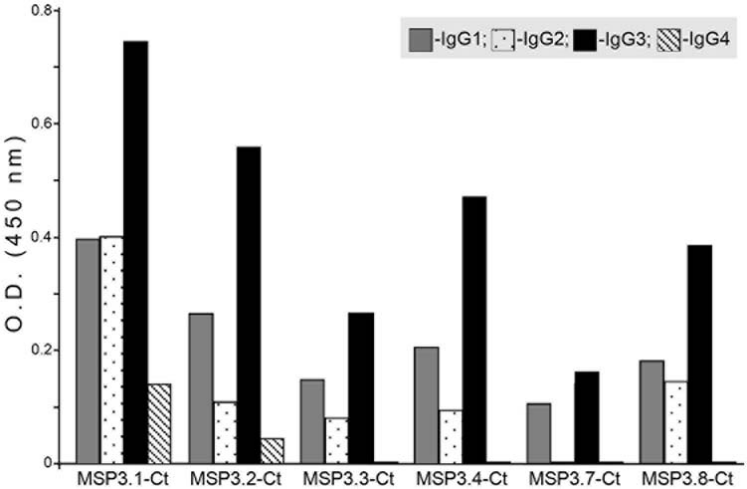
Pattern of antibody subclass reactivity observed against different members of the MSP3-family of proteins in a pool of hyperimmune sera from malaria endemic village Dielmo, Senegal. The histograms represent mean O.D._450_ values obtained after subtracting the reactivity against BSA.

The striking similarities in sequence organization observed between the C-terminal halves of the MSP3-family members together with limited diversity observed for the regions identified as targets of parasite killing antibodies in MSP3.1 and MSP3.2 (Fig. 1B), led us to investigate the antigenic relatedness between these proteins. The related C-terminal half of each member was expressed as recombinant His-tag proteins in *E. coli* (Table S2). A pool of malaria hyperimmune sera (from Dielmo, Senegal) showed distinct levels of reactivity against the six C-terminal recombinant proteins (Fig. 5). The patterns of IgG subclass distribution against each recombinant protein indicated differences in their antigenic characteristics.

Human antibodies affinity-purified against each of the C-terminal recombinant proteins were found to display varying degrees of cross-reactivity with the remaining members of the family, as determined by ELISA (Table 2). While anti-MSP3.1-Ct antibodies exhibited the lowest extent of cross-reactivity to other members of the family, MSP3.1-Ct itself appeared to be the target most widely recognized by antibodies affinity-purified on other members of the family. The opposite situation was found for MSP3.4-Ct. Although anti-MSP3.4-Ct antibodies displayed the highest level of cross-reactivity to other members of the family, MSP3.4-Ct itself was less well recognized by antibodies affinity-purified on other members. Antibodies against the remaining members of the family displayed intermediate levels of cross-reactivity. None of the naturally occurring antibody preparation affinity-purified against the C-terminal recombinants displayed reactivity against a set of negative control antigens which included unrelated recombinant malaria antigen DG571-His and BSA.

**Table 2:** Naturally occurring antibodies against related C-terminal regions of the MSP3 family of proteins exhibit cross-reactivity.

	MSP3.1-Ct	MSP3.2-Ct	MSP3.3-Ct	MSP3.4-Ct	MSP3.7-Ct	MSP3.8-Ct	DG571-His	BSA
**anti-MSP3.1-Ct**	0.156 **(100%)**	0.009 **(5.8%)**	0.052 **(33.3%)**	0.007 **(4.5%)**	0.054 **(34.6%)**	0.036 **(23.1%)**	0.005 **(3.2%)**	0.006 **(3.8%)**
**anti-MSP3.2-Ct**	0.073 **(54.5%)**	0.134 **(100%)**	0.029 **(21.6%)**	0.005 **(3.7%)**	0.052 **(38.8%)**	0.05 **(37.3%)**	0.005 **(3.7%)**	0.006 **(4.5%)**
**anti-MSP3.3-Ct**	0.135 **(117.4%)**	0.052 **(45.2%)**	0.115 **(100%)**	0.006 **(5.2%)**	0.115 **(100%)**	0.054 **(47.0%)**	0.006 **(5.2%)**	0.006 **(5.2%)**
**anti-MSP3.4-Ct**	0.158 **(216.4%)**	0.032 **(43.8%)**	0.095 **(130.1%)**	0.073 **(100%)**	0.107 **(146.6%)**	0.075 **(102.7%)**	0.007 **(9.6%)**	0.007 **(9.6%)**
**anti-MSP3.7-Ct**	0.047 **(32.0%)**	0.006 **(4.1%)**	0.005 **(3.4%)**	0.005 **(3.4%)**	0.147 **(100%)**	0.007 **(4.8%)**	0.006 **(4.1%)**	0.006 **(4.1%)**
**anti-MSP3.8-Ct**	0.085 **(72.6%)**	0.027 **(23.1%)**	0.031 **(26.5%)**	0.009 **(7.7%)**	0.062 **(53.0%)**	0.117 **(100%)**	0.007 **(6.0%)**	0.006 **(5.1%)**

Antibodies affinity-purified against C-terminal recombinant protein from each member of MSP3-family of proteins were assessed for their cross-reactivity towards other members by ELISA. O.D._450_ values obtained for the reactivity of affinity-purified antibodies towards each recombinant protein are shown. The shaded boxes represent reactivity of the antibodies affinity-purified against their respective recombinant proteins, which was considered to be 100%. The degree of cross-reactivity towards other members of the family is expressed as fractions of 100%, shown in bold.

**Figure 6:**
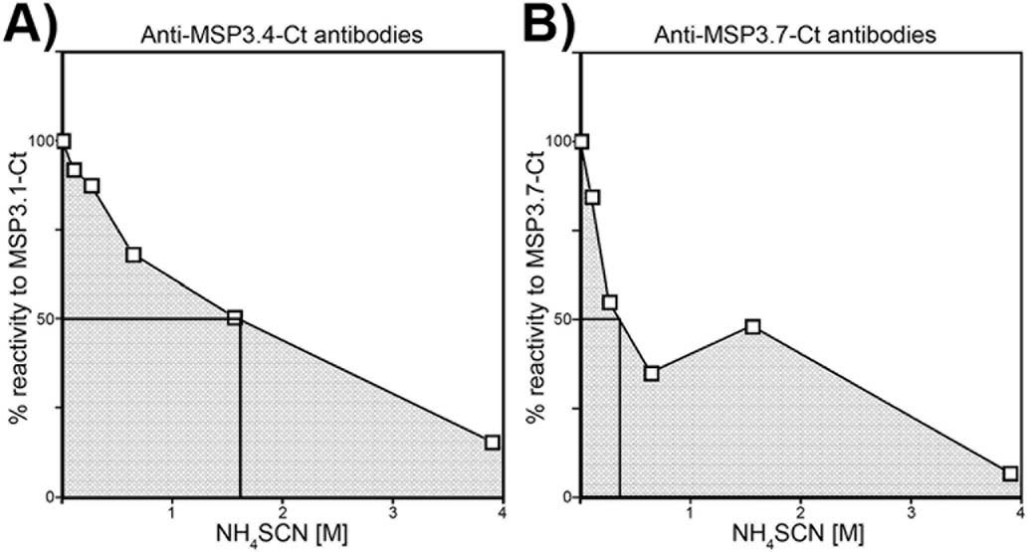
Graphical presentation of antibody binding avidity against members of the MSP3-family of proteins under increasing concentrations of NH_4_SCN. Shown here are two examples of affinity-purified antibodies *Panel* A: reactivity of anti-MSP3.4-Ct antibodies against MSP3.1-Ct and *Panel* B: reactivity of anti-MSP3.7-Ct antibodies towards itself. The measure of antigen-antibody reactivity in absence of NH_4_SCN was considered to be 100%, and the reactivity obtained in presence of increasing concentrations of NH_4_SCN was expressed as fractions of that 100%. Since the antibody binding did not display a linear relationship with increasing concentrations of the chaotropic salt, antibody binding avidity was determined by calculating the ‘% area covered by each curve’, as represented by the shaded area in the figure.

**Figure 7:**
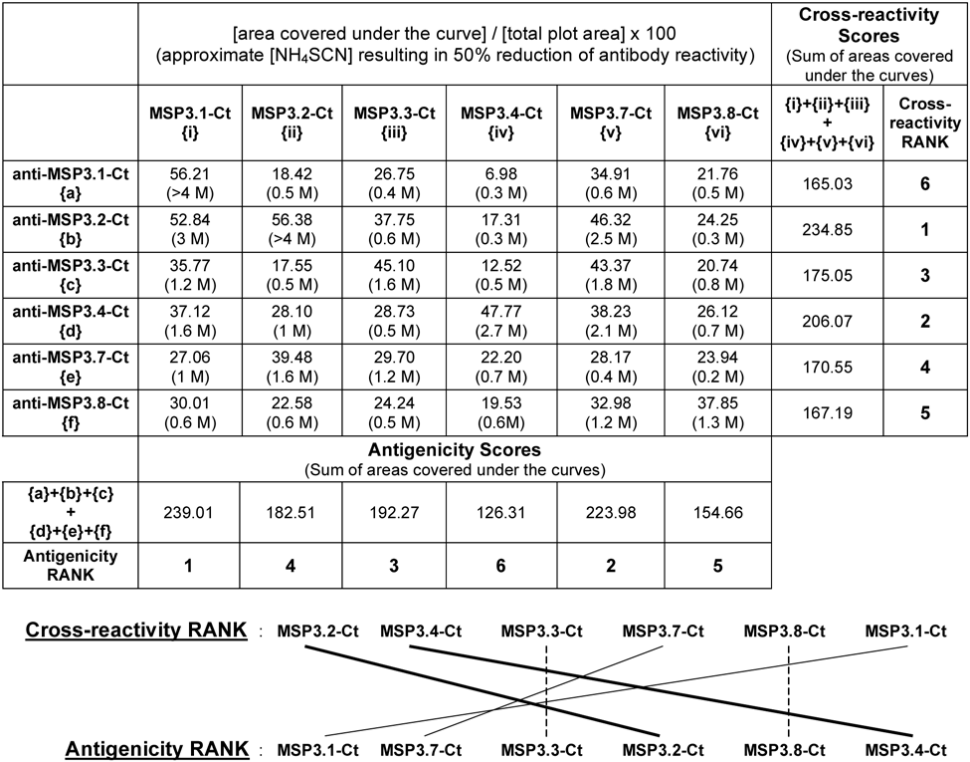
Binding avidity study of antibody cross-reactivity observed amongst the related regions of the MSP3-family of proteins. Antibody binding avidity for each antigen-antibody reaction is expressed in terms of ‘% area covered by the curve’ (as explained in the text and Figure 6). Summing the binding avidities displayed by different antibody-preparations towards any given antigen provides an estimate of its ‘antigenicity’, obtained here along the columns. Similarly, summing the binding avidities displayed by any antibody-preparation towards different antigens provides an estimate about the degree of ‘cross-reactivity’ for that antibody preparation, obtained here across the rows. Arranging the molecules in the order of their increasing ‘antigenicity’ and the degree of ‘cross-reactivity’ displayed by affinity-purified antibodies shows MSP3.1-Ct to be the most while MSP3.4-Ct being the least antigenic molecules in the family. Conversely, anti-MSP3.1-Ct antibodies displayed least cross-reactivity in contrast to higher degree of cross-reactivity displayed by anti-MSP3.2-Ct and anti-MSP3.4-Ct antibodies.

In order to better characterize the above cross-reactivities, the binding avidity of the affinity-purified antibodies was investigated by ELISA under increasing concentrations of the chaotropic ion, ammonium thiocyanate (NH_4_SCN). Various patterns of avidity were observed for each affinity-purified antibody against different members of the family. Moreover, for several antigen-antibody combinations, the multiple slopes observed indicate a mix of antibody species with distinct binding avidities (an example is shown in Fig. 6). The area under the curve (AUC) was used to estimate the overall binding avidity of these heterogeneous populations of naturally occurring antibodies for the different MSP3-family members. The summation of the AUCs in turn allowed an estimation of “antigenicity” (the ability of any MSP3-family protein to be recognized by antibodies against other family proteins), and of “cross-reactivity” (the reactivity of protein-specific antibodies with other MSP3 proteins). In Figure 7, ‘antigenicity ranking’ is obtained by summation along the columns, whereas the ‘cross-reactivity ranking’ is obtained by summation along the rows. Through such analysis, recombinant MSP3.1-Ct was found to be the ‘most antigenic’ and MSP3.4-Ct the ‘least antigenic’. Anti-MSP3.1-Ct antibodies were found to be the least cross-reactive, whereas anti-MSP3.4-Ct and anti-MSP3.2-Ct antibodies were the most cross-reactive. These results suggest that a complex network of cross-reactivity exists amongst naturally occurring antibodies against different members of the MSP3 family of proteins.

**Figure 8:**
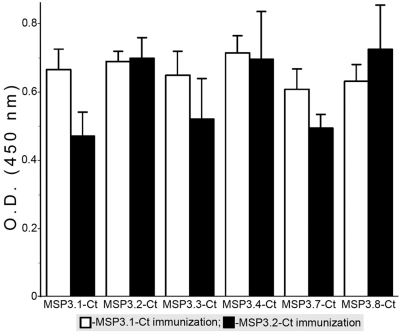
Immunization in mice generates antibodies cross-reactive against the related regions of MSP3-family of proteins. Two groups Balb/C mice (5 mice/group), were immunized with the related C-terminal recombinant proteins from MSP3.1 and MSP3.2, with montanide as adjuvant. The histograms show mean O.D._450_ values obtained for the reactivity of anti-MSP3.1-Ct and anti-MSP3.2-Ct mice sera against different members of the MSP3-family of proteins. The error-bars represent s.d. values.

Immunization of mice with MSP3.1-Ct and MSP3.2-Ct recombinant proteins also generated antibodies cross-reactive to other members of the MSP3-family (Fig. 8). In contrast to the low degree of cross-reactivity displayed by naturally occurring anti-MSP3.1-Ct antibodies, immunization of mice generated anti-MSP3.1-Ct antibodies which displayed a wider pattern of cross-reactivity, *i.e.* comparable to that of anti-MSP3.2-Ct antibodies. The differences between the fine specificity of antibody resulting from natural exposure of from artificial immunization deserve further investigations. It is however, encouraging for vaccine development to note that the network of antibody cross-reactivity amongst the MSP3-proteins, which develops through natural exposure, can be mimicked by antibodies elicited by artificial immunization.

### The 6 MSP3-proteins are targets of antibodies effective in ADCI

**Figure 9:**
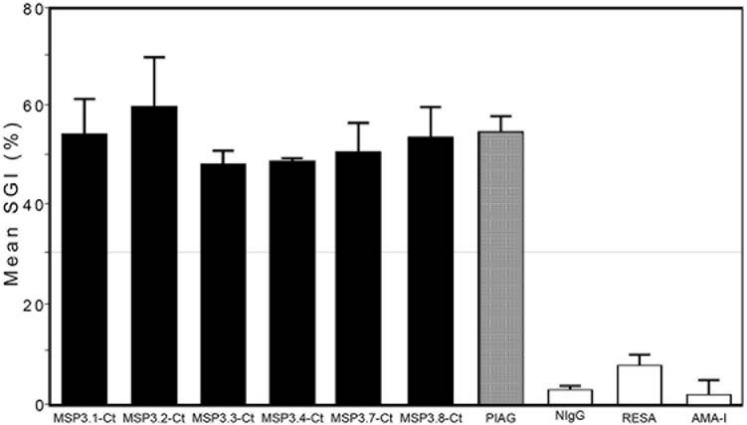
Effect of human antibodies affinity-purified against the related C-terminal region of the MSP3 family of proteins on parasite growth in ADCI assay. The histograms represent mean values of % SGI (as explained in the text) from two independent experiments±standard error; values of >30% are significant. PIAG, positive control IgG from the pool of Ivory Coast adult sera used for passive transfer in humans [6]. NIgG, negative control IgG from a pool of Caucasian adult sera, never exposed to malaria infection. Antibodies affinity purified against two other malaria antigens RESA and AMA-I did not show significant parasite inhibition at the concentrations used in this assay.

Anti-MSP3.1-Ct and anti-MSP3.2-Ct antibodies were found earlier to mediate the monocyte dependent inhibition of *P. falciparum* growth [19], [20]. Antibodies affinity-purified from hyperimmune sera against the C-terminal regions of the six related members of the MSP3-family, were adjusted to an equal effective concentration, *i.e.* yielding the same reactivity to the native parasite protein as determined by IFAT. Affinity-purified antibodies against each MSP3-family member were found able to mediate a strong monocyte dependent parasite inhibition in ADCI assay *in vitro* (Fig. 9). The inhibition levels were of magnitude similar to that obtained using the pool of African IgG (PIAG) effective in passive transfer in humans [6]. Results demonstrate that each member of the MSP3-family of proteins serves as a target for naturally occurring antibodies with anti-parasite effect, which is in accordance with their sequence similarities and the network of cross-reactivity displayed by antibodies against them.

## Discussion

In the present work, we have characterized a new multi-gene family coding for six merozoite surface proteins that share the same structural organization of their C-terminal regions and have attractive immunological features. These genes are located contiguously on the same locus of chromosome 10, and share the NLRNA/NLRNG signature motif [21] besides homologous C-terminal regions. These C-terminal regions bear particular importance. Although they show considerable divergence between one paralog and any of the others, yet each paralog is remarkably well-conserved from one isolate to another and in the process share epitopes that generate a wide network of cross-reactive antibodies with all six members being the targets of antibodies mediating parasite killing through ADCI.

Four members of the *P. falciparum MSP3*-family, *MSP3.1*, *MSP3.2*, *MSP3.3* and *MSP3.7* (originally described as *MSP3*, *MSP6*, *H101* and *H103* respectively), had been previously identified as merozoite surface proteins [9], [30], [31]. The family is actually made of eight ORFs, which had been detected previously through *P. falciparum* genome analysis [32], but among which we were led to exclude two ORFs because they do not share the same C-terminus organization, have absent or unstable transcription and debatable protein expression. We therefore propose to group the remaining six MSP3-related proteins based on their sequence relatedness and antigenic cross-reactivity as constituting the *P. falciparum* MSP3 family of proteins.

This family has similarities with the MSP3 orthologs described in different malaria parasite species such as *P. knowlesi* and *P. vivax* in which multi-allelic homologs have been described [21], [22], [40], [41], as is now the case for *P. falciparum*. All known *MSP3*-orthologs across different species are characterized by the N-terminal signature motif present shortly after the predicted signal peptide sequence [21]. However, although they do share the same similar structural organization in their C-terminal regions between species and paralogs, and are as well-conserved from one isolate to the other [42], [43] as we find in *P. falciparum*, the *P. knowlesi* and *P. vivax* MSP3 orthologs also markedly differ from the six *P. falciparum* MSP3 genes. Each of the MSP3 orthologs and paralogs in *P. vivax* and *P. knowlesi* have an extensive central alanine-rich coiled-coil domain similar to the shorter coiled-coil region found only in the *P. falciparum* MS3.1 paralog. All other *P. falciparum* family members have in each other, ‘unique’ domains that are very different from one another.

The *P. falciparum MSP3*-multigene family differs from other known *P. falciparum* gene families expressed during the development of asexual blood stages. Members of the antigenic variation super gene families such as the *var*, *rifin* and *stevor*, which show multi-exon organization, are dispersed mostly in the recombinogenic subtelomeric regions of different chromosomes and, for the most part, display mutually exclusive gene expression patterns. In contrast, MSP3-family genes are structured as a single exon with a contiguous organization on the same chromosome and importantly, all are expressed simultaneously. The MSP3 multi-gene family also differs from other multi-gene families encoding for merozoite secretory organelle proteins such as the *RBL*, *EBL* and *RAP* that have multi-exon gene structure and exhibit considerable variability in gene expression, as well as redundancy in functionality [33]–[35]. Though members of other gene families share a gene organization in common, throughout the family they are usually quite diverse in their amino-acid sequences and the resulting epitopes are generally not cross-reactive, except for the rare exceptions [36]. For example, naturally occurring antibodies against the EGF-like domains of different MSPs are not cross-reactive [37], [38]. In contrast, the C-terminal regions of the MSP3 family of proteins, while related in their overall organization, exhibit intergenic diversity, but also contain polypeptide sequences that generate shared or cross-reactive epitopes, which in contrast with other multi-gene families are highly conserved across parasite isolates.

Gene knockout approaches are currently considered as one means to determine the functional importance of a given gene in parasite biology. This was actually applied to several members of the MSP3-family :*ΔMSP3.1* [39] and *ΔMSP3.2* (Mills & Cowman, *personal communication*), *ΔMSP3.3* and *ΔMSP3.7* [31]. However, the individual gene disruptions caused little or no effect on the *in vitro* survival of mutant parasites. Perhaps one reason for these apparently paradoxical findings may lie in the fact that all genes of the MSP3 family are transcribed together with the corresponding proteins simultaneously expressed on the surface of *P. falciparum* merozoites. Paralogous structures expressed by the remaining members of the MSP3 family could thus compensate for the loss of either one individually, providing the parasite with functional “spare-wheels”, whatever their as yet, undescribed biological function may be. On the other hand, these paralogs except for their C-terminal regions have radically different domain structures in the remainder of the proteins implying, perhaps, differing functional roles for each. The lack of an evident phenotype *in vitro* upon gene disruption may, alternatively, imply that a phenotype can only be revealed *in vivo* through host-parasite interactions, perhaps that involve the ‘conserved’ C-terminal domains.

The related C-terminal regions from each member of the family were found to be targeted by antibodies that inhibit parasite growth in cooperation with monocytes, with a magnitude similar to the inhibition original found to be mediated by native antibodies recognizing the MSP3.1 protein. This broadens the scope of antigens involved in naturally acquired protection and indicates that vaccine constructs based on them would likely be valuable. Detailed analyses of MSP3.1, both on epidemiological and clinical grounds as well as in assays reflecting antibody mediated protection, led to the identification of three regions as targets for protective antibodies [12]. The highest regions of homology were found within these related C-terminal parts of the MSP3 proteins, but the various MSP3 genes also showed substantial diversity in sequence from one gene to the other. The detailed antigenic analysis of the MSP3 family members showed that, despite differences among their primary amino-acid sequences, some epitopes and their antigenic properties are sufficiently conserved to generate cross-reactive antibodies. The extent of cross-reactivity is such that when an antibody response in humans is detected against one member of the family these same antibodies could have been elicited by any other member of the multi-gene family. This results in a large network of reactivities for each specific antibody across each of the members of the family; certainly with demonstrable variations in regards to avidity.

Polymorphism in malaria parasite genes generated by random mutations is a frequent characteristic of many genes coding for antigens. This polymorphism is thought to have emerged under selective immune pressure that frequently favors epitopic regions involved in protection. Therefore, polymorphism selected by immunity is considered a major bottleneck for vaccine development that can lead to strain-specific protection or immune evasion. This widely accepted notion differs markedly in the case of the MSP3 multi-gene family as in this instance the evolutionary drive responsible for sequence diversity cannot result from immune selection of random mutations. Whereas the N-terminal regions of these genes are highly divergent, the C-terminal portions display limited sequence diversity between strains and share high similarity in sequence and general structure as well as antigenic characteristics. In addition, the full sequence conservation of the C-terminal regions of each member of the MSP3 family among distinct isolates, resulting in a conservation of differences between genes, is striking and most unusual. We speculate that *in vivo* the C-terminal parts of the proteins function at the interface of the parasite with the immune responses of the host. The anti-parasite effect of antibodies against each C-terminal region together with the antigenic relatedness shared between all members, suggests a minimal selective advantage to random mutations, as antibodies generated against any one member could still cross-react to other non-mutated member and mediate parasite killing. In the context of a compromise between the immunized host and infecting parasite, the nearly full conservation of both the homology and diversity observed among the C-terminal regions of the MSP3 family of proteins lead us to raise two main hypotheses:

The first hypothesis is that this diversity generates a wider range of antibody specificities reactive with the related members of an antigenic network than would that of a single antigen. A wider range of diversity in affinity, avidity and fine-specificity in the antibody repertoire would result in reactivity to the wide range of original and related epitopes and lead to greater efficacy of the antibody mediated monocyte-dependent parasite killing.

The second hypothesis is that the differences in the sequence also provide T-helper cell epitope diversity, resulting in the induction of antibody responses in a wider range of human genetic backgrounds, each gene sequence being better fitted to a given MHC class-II subset. Hence, in this case, the conservation of the diversity could provide T-cell help leading to the generation of the same essential antibodies directed to the conserved cross-reactive B-cell epitopes in most individuals. The results obtained in a MSP3.1 vaccine trial are in support of this hypothesis as not all volunteers immunized with only one member of the family, MSP3.1, developed antibodies reactive with native parasite proteins, whereas in endemic areas most individuals develop antibodies to those main B-cell epitopes [10], [12], [17], [44].

These two hypotheses are not mutually exclusive, as the first one concerns the regions of highest homology, the cross-reactive B-cell epitopes, and the second the regions of greatest divergence among the C-term regions, which were found to define T helper-cell epitopes in MSP3.1. In both cases, the results point to the production of anti-MSP3 cross-reactive antibodies that, by triggering ADCI, have the ability to substantially control parasite multiplication and in the process maintain low to moderate parasite densities in the human host. This conservation of diversity would also generate immune responses that can be thought of as ensuring the “objective” of successful parasites; survival of both the host and the parasite.

Our observations and findings enlarge the present perspectives on the function of merozoite surface antigens and have fundamental implications for the nature of the host-parasite interaction, as may pertain to maintaining homeostasis between *P. falciparum* and human beings. There may also be practical consequences for vaccine development as our results suggest that an improved MSP3 vaccine should combine the other various C-terminus regions in the family in order to generate a wider range of antibodies reacting with each of the various MSP3 family antigens to improve the vaccine immunogenicity in humans across most genetic backgrounds.

## Supporting Information

Table S1 (0.20 MB PDF)

Table S2 (0.16 MB PDF)

Text S1 Supporting Info Sequence conservation
(0.22 MB PDF)

## References

[1] McGregor IA, Wilson RJM, Wernsdorfer WH, McGregor I (1988). Specific immunity acquired in man.. Malaria: Principles and practice of malariology.

[2] Sergent ED, Parrot L (1935). L'immunité, la prémunition et la résistance innée.. Archives de l'institut Pasteur d'Algérie TXIII.

[3] Cohen S, Mc Gregor A, carrington S (1961). Gamma globulin and acquired immunity to human malaria.. Nature.

[4] Edozien JC, Gilles HM, Udeozo IOK (1962). Adult and cord blood gammaglobulin and immunity to malaria in Nigerians.. Lancet.

[5] McGregor IA, Carrington SP, Cohen S (1963). Treatment of East African *P. falciparum* with West African human gammaglobulin.. Trans Roy Soc Trop Med Hyg.

[6] Sabchareon A, Burnouf T, Ouattara D, Attanath P, Bouharoun-Tayoun H (1991). Parasitologic and clinical human response to immunoglobulin administration in *falciparum* malaria.. Am J Trop Med Hyg.

[7] Khusmith S, Druilhe P (1983). Cooperation between antibodies and monocytes that inhibit *in vitro* proliferation of *Plasmodium falciparum*.. Infec Immun.

[8] Bouharoun-Tayoun H, Attanah P, Sabchareon A, Chongsuphajaisiddhi T, Druilhe P (1990). Antibodies that protect humans against *Plasmodium falciparum* blood stages do not on their own inhibit parasite growth and invasion *in vitro*, but act in cooperation with monocytes.. J Exp Med.

[9] Oeuvray C, Bouharoun-Tayoun H, Gras-Masse H, Bottius E, Kaidoh T (1994). Merozoite surface protein-3: a malaria protein inducing antibodies that promote *Plasmodium falciparum* killing by cooperation with blood monocytes.. Blood.

[10] Roussilhon C, Oeuvray C, Muller-Graf C, Tall A, Rogier C (2007). Long-term clinical protection from *falciparum* malaria is strongly associated with IgG3 antibodies to merozoite surface protein 3.. PLoS Med.

[11] Soe S, Theisen M, Roussilhon C, Aye KS, Druilhe P (2004). Association between protection against clinical malaria and antibodies to merozoite surface antigens in an area of hyperendemicity in Myanmar: complementarity between responses to merozoite surface protein 3 and the 220-kilodalton glutamate-rich protein.. Infect Immun.

[12] Singh S, Soe S, Mejia JP, Roussilhon C, Theisen M (2004). Identification of a conserved region of *Plasmodium falciparum* MSP3 targeted by biologically active antibodies to improve vaccine design.. J Infect Dis.

[13] Polley SD, Tetteh KK, Lloyd JM, Akpogheneta OJ, Greenwood BM (2007). *Plasmodium falciparum* merozoite surface protein 3 is a target of allele-specific immunity and alleles are maintained by natural selection.. J Infect Dis.

[14] Osier FH, Polley SD, Mwangi T, Lowe B, Conway DJ (2007). Naturally acquired antibodies to polymorphic and conserved epitopes of *Plasmodium falciparum* merozoite surface protein 3.. Parasite Immunol.

[15] Jafarshad A, Dziegiel MH, Lundquist R, Nielsen LK, Singh S (2007). A novel antibody-dependent cellular cytotoxicity mechanism involved in defense against malaria requires costimulation of monocytes FcgammaRII and FcgammaRIII.. J Immunol.

[16] Hisaeda H, Saul A, Reece JJ, Kennedy MC, Long CA (2002). Merozoite surface protein 3 and protection against malaria in *Aotus nancymai* monkeys.. J Infect Dis.

[17] Druilhe P, Spertini F, Soe Soe D, Corradin GP, Mejia P (2005). A malaria vaccine that elicits antibodies able to kill *P. falciparum*.. PLoS Med.

[18] Sirima SB (2008). A double blind randomized controlled dose escalation Phase Ib field trial in 12–24 months old children in Burkina Faso to evaluate the safety and immunogenicity of the *Plasmodium falciparum* merozoite surface protein-3 LSP adjuvanted in aluminium hydroxyde versus Engerix B.. Am J Trop Med Hyg.

[19] Lusingu J (2008). A phase Ib study of the safety of MSP3-LSP candidate malaria vaccine in Tanzanian children aged 12–24 months.. Am J TropMed Hyg.

[20] Singh S, Soe S, Roussilhon C, Corradin G, Druilhe P (2005). *Plasmodium falciparum* merozoite surface protein 6 displays multiple targets for naturally occurring antibodies that mediate monocyte-dependent parasite killing.. Infect Immun.

[21] Galinski MR, Ingravallo P, Corredor-Medina C, Al-Khedery B, Povoa M (2001). *Plasmodium vivax* merozoite surface proteins-3beta and-3gamma share structural similarities with *P. vivax* merozoite surface protein-3alpha and define a new gene family.. Mol Biochem Parasitol.

[22] David PH, Hudson DE, Hadley TJ, Klotz FW, M LH (1985). Immunization of monkeys with a 140 kilodalton merozoite surface protein of *Plasmodium knowlesi* malaria: appearance of alternate forms of this protein.. J Immunol.

[23] Lupas A, Van Dyke M, Stock J (1991). Predicting coiled coils from protein sequences.. Science.

[24] Wolf E, Kim PS, Berger B (1997). MultiCoil: a program for predicting two- and three-stranded coiled coils.. Protein Sci.

[25] Bornberg-Bauer E, Rivals E, M V (1998). Computational approaches to identify leucine zippers.. Nucleic Acids Res.

[26] Bouharoun-Tayoun H, Druilhe P (1992). *Plasmodium falciparum* malaria: evidence for an isotype imbalance which may be responsible for delayed acquisition of protective immunity.. Infect Immun.

[27] Druilhe P, Bouharoun-Tayoun H (2002). Human antibody subclass ELISA.. Methods Mol Med.

[28] Bouharoun-Tayoun H, Attanath P, Sabchareon A, Chongsuphajaisiddhi T, P. D (1990). Antibodies that protect humans against *Plasmodium falciparum* blood stages do not on their own inhibit parasite growth and invasion in vitro, but act in cooperation with monocytes.. J Exp Med.

[29] McColl DJ, Silva A, Foley M, Kun JF, Favaloro JM (1994). Molecular variation in a novel polymorphic antigen associated with *Plasmodium falciparum* merozoites.. Mol Biochem Parasitol.

[30] Trucco C, Fernandez-Reyes D, Howell S, Stafford WH, Scott-Finnigan TJ (2001). The merozoite surface protein 6 gene codes for a 36 kDa protein associated with the *Plasmodium falciparum* merozoite surface protein-1 complex.. Mol Biochem Parasitol.

[31] Pearce JA, Mills K, Triglia T, Cowman AF, Anders RF (2005). Characterisation of two novel proteins from the asexual stage of *Plasmodium falciparum*, H101 and H103.. Mol Biochem Parasitol.

[32] Cowman AF, BS C (2002). The *Plasmodium falciparum* genome–a blueprint for erythrocyte invasion.. Science.

[33] Reed MB, Caruana SR, Batchelor AH, Thompson JK, Crabb BS (2000). Targeted disruption of an erythrocyte binding antigen in *Plasmodium falciparum* is associated with a switch toward a sialic acid-independent pathway of invasion.. Proc Natl Acad Sci U S A.

[34] Kaneko O, Fidock DA, Schwartz OM, M LH (2000). Disruption of the C-terminal region of EBA-175 in the Dd2/Nm clone of *Plasmodium falciparum* does not affect erythrocyte invasion.. Mol Biochem Parasitol.

[35] Duraisingh MT, Triglia T, Ralph SA, Rayner JC, Barnwell JW (2003). Phenotypic variation of *Plasmodium falciparum* merozoite proteins directs receptor targeting for invasion of human erythrocytes.. EMBO J.

[36] Chattopadhyay R, Sharma A, Srivastava VK, Pati SS, Sharma SK (2003). *Plasmodium falciparum* infection elicits both variant-specific and cross-reactive antibodies against variant surface antigens.. Infect Immun.

[37] Black CG, Wang L, Hibbs AR, Werner E, RL C (1999). Identification of the *Plasmodium chabaudi* homologue of merozoite surface proteins 4 and 5 of *Plasmodium falciparum*.. Infect Immun.

[38] Black CG, Wang L, Wu T, RL C (2003). Apical location of a novel EGF-like domain-containing protein of *Plasmodium falciparum*.. Mol Biochem Parasitol.

[39] Mills KE, Pearce JA, Crabb BS, Cowman AF (2002). Truncation of merozoite surface protein 3 disrupts its trafficking and that of acidic-basic repeat protein to the surface of *Plasmodium falciparum* merozoites.. Mol Microbiol.

[40] Pain A, Böhme U, Berry AE, Mungall K, Finn RD (2008). The genome of the simian and human malaria parasite *Plasmodium knowlesi*.. Nature.

[41] Carlton JM, Adams JH, Silva JC, Bidwell SL, Lorenzi H (2008). Comparative genomics of the neglected human malaria parasite *Plasmodium vivax*.. Nature.

[42] Rayner JC, Corredor V, Feldman D, Ingravallo P, Iderabdullah F (2002). Extensive polymorphism in the *Plasmodium vivax* merozoite surface coat protein MSP-3alpha is limited to specific domains.. Parasitology.

[43] Rayner JC, Huber CS, Feldman D, Ingravallo P, Galinski MR (2004). *Plasmodium vivax* merozoite surface protein PvMSP-3 beta is radically polymorphic through mutation and large insertions and deletions.. Infect Genet Evol.

[44] Audran R, Cachat M, Lurati F, Soe S, Leroy O (2005). Phase I malaria vaccine trial with a long synthetic peptide derived from the merozoite surface protein 3 antigen.. Infect Immun.

