# Supplementary material for: A Conserved Multi-Gene Family Induces Cross-Reactive Antibodies Effective in Defense against Plasmodium falciparum
Source: PLoS One. 2009 Apr 30;4(4):e5410. doi: 10.1371/journal.pone.0005410 (PMC2671155; doi:10.1371/journal.pone.0005410)
Supplement: Table S1 — (0.20 MB PDF) [file pone.0005410.s001.pdf]

Sequence	Oligonucleotide primer pairs used for PCR amplifications	Amino acid sequences of the unique regions (numbers show a.a. positions of the 3D7sequences)
**MSP3.1 unique**	F: 5’-CGCAAGATCTGGTTATACGGAAGAATTAAAAGC-3 ’<br>R: 5’-CGCACCATGGCTATGAAGATTTTTCAGCATCATC-3 ’	71-GYTEELKAKKASEDAEKAANDAENASKEAEEAAKEAVNL<br>KESDKSYTKAKEACTAASKAKKAVETALKAKDDAEKSS-147
**MSP3.2 unique**	F: 5’-CGCAAGATCTACATCAAGGAGGAAATAATGTAATTCC-3 ’<br>R: 5’-CGCACCATGGCTAATTATTATTCAGAGAAGTTGTAG-3 ’	112-TSGGNNVIPLPIKQSGENQYTVTSISGIQKGANGLTGATEN<br>ITQVVQANSETNKNPTSHSNSTTTSLNNN-181
**MSP3.3 unique**	F: 5’-CGCAAGATCTATTTATGAAACTACAGGAAGTCTAAGG-3 ’<br>R: 5’-CGCACCATGGCTAATCATTTTCTAAACTACTATCAG-3 ’	72-IYETTGSLGTGVESVKAIDGESGTSMDSKPKENKISTEPGA<br>DQVSIGLVNESDSSLEND-130
**MSP3.4 unique**	F: 5’-CGCAAGATCTGATTCTCTAACAACCACTTCTTTATCAACG-3 ’<br>R: 5’-CGCACCATGGCTAATTATTGTTGTAGTTATTATTTCC-3 ’	459-DSLTTTSLSTSINSVRDSSNLDQRGNITTSQGNSHRATVVQ<br>QVDQTNRLDNVNSVTQRGNNNYNNN-524
**MSP3.5 unique**<br>(odd member)	F: 5’-CGCAAGATCTCAATCCAAAGGAAATAGTGGTACTAAGG-3 ’<br>R: 5’-CGCACCATGGCTAATCTAAGTATATATTATTGTCG-3 ’	210-QSKGNSGTEGDGSSVFGSIFGSLLTPIDSLLEKFIGSNNTN<br>SDSNVKNTSMGNGQNKYDNN IYLD-274
**MSP3.6 unique**<br>(odd member)	F: 5’-CGCAAGATCTCTTGATATCTTTTACT-3 ’<br>R: 5’-CGCACCATGGCTAACCTATTTCAGTTTCCG-3 ’	95-LDIFTENKEQKNEEVPMKIEVVNDGEEVKTEYVSEKNEEV<br>ENKSETEIG-143
**MSP3.7 unique**	F: 5’-CGCAAGATCTTATGAAGCTTCAGAATATATAGA-3 ’<br>R: 5’-CGCACCATGGCTACCCAGTACCTACAAATATACC-3 ’	60-YEASEYIEKQNDILNMYNDEKEKNNNNSLDTNVTKNTVID<br>NSNKFQSIEDNNVYNKGIFVGTG-122
**MSP3.8 unique**	F: 5’-CGCAAGATCTGTGAGTAATAGTGTGAATGCCTTACC-3 ’<br>R: 5’-CGCACCATGGCTAGCTACCTTGGTTTACTTCTTGG-3 ’	475-VSNSVNALPEPGQITLPDPSLKQTTQQENQPVVETPVTTA<br>VINEHQGQTEPNKGDNNNERENHESNVGSIQEVNQGS-551

Table S1: Pairs of primer pairs used for cloning the unique region sequences. The column on the right shows amino acid sequences of the unique regions. The amino-acids have been numbered with respect to the 3D7 sequence.
