# Supplementary material for: A Conserved Multi-Gene Family Induces Cross-Reactive Antibodies Effective in Defense against Plasmodium falciparum
Source: PLoS One. 2009 Apr 30;4(4):e5410. doi: 10.1371/journal.pone.0005410 (PMC2671155; doi:10.1371/journal.pone.0005410)
Supplement: Table S2 — (0.16 MB PDF) [file pone.0005410.s002.pdf]

Sequence	Oligonucleotide primer paiars used for PCR amplifications	Amino acid sequences for C-term (numbers show a.a. positions in 3D7)
**MSP3.1CT**	F: 5'-CGCAAGATCTTATGAAAAGGCAAAAAATGCT-3 '<br>R: 5'-CGCACCATGGTTAATGATTTTTAAAATATTTGGA-3 '	167-YEKAKNAYQKANQAVLKAKEASSY …<br>……GNNQIDSTLKDLVEELSKYFKNH-371
**MSP3.2CT**	F: 5'-CGCAAGATCTTCTGAAACAAATAAAAATCCTACTTCTCAT-3 '<br>R: 5'-CGCACCATGGTTAATTATTACTAAATAGATGGATCATTTCTTG-3 '	161-SETNKNPTSHSNSTTTSLNNNILGWE…<br>…...NEKNEIDSTINNLVQEMIHLFSNN-371
**MSP3.3CT**	F: 5'-CGCAAGATCTTATGAGAAGAAAAATGAAAATA-3 '<br>R: 5'-CGCACCATGGTTAATTATATGTAAAAAATTCCAT-3 '	228-YEKKNENKNVSNVDSKTKSNEKGR…<br>..…LNGKNELDATIRRLKHRFMEFFTYN-424
**MSP3.4CT**	F: 5'-CGCAAGATCTGATAATGTAAACTCTGTAACG-3 '<br>R: 5'-CGCACCATGGTTATTTTTGAAATAAATCTGTCAT-3 '	508-DNVNSVTQRGNNNYNNNLERGLGS…<br>...…FNDNNNLETIFKGLTEDMTDLFQK-697
**MSP3.7CT**	F: 5'-CGCAAGATCTCCTGAAGGACCAAGAGCAAA-3 '<br>R: 5'-CGCACCATGGTCAATAGTTATTTAAAAAAAAAGT-3 '	214-PEGPRANNRNENNQNTDPYNHYFA…<br>...…QTNNQLDPSLKDLENELTFFLNNY-405
**MSP3.8CT**	F: 5'-CGCAAGATCTCATGAAAGTAATGTTGGTAG-3 '<br>R: 5'-CGCACCATGGTTAATTTTTAAATAAATTTGTAAT-3 '	537-HESNVGSIQEVNQGSVSEESHSKTI…<br>...…LEEGNGSDSTLNSLSKDITNLFKN-762

Table S2: Pairs of primer pairs used for cloning the related carboxy-terminal regions, from each member of the MSP3-family of proteins. The column on the right shows amino acid sequences of the C-term regions. The amino-acids have been numbered with respect to the 3D7 sequence. The related caroxy-terminal recombinants were not designed from MSP3.4 and MSP3.5 as these sequences do not share sequence relatedness with other members of the family.
