# Supplementary material for: A Conserved Multi-Gene Family Induces Cross-Reactive Antibodies Effective in Defense against Plasmodium falciparum
Source: PLoS One. 2009 Apr 30;4(4):e5410. doi: 10.1371/journal.pone.0005410 (PMC2671155; doi:10.1371/journal.pone.0005410)
Supplement: Text S1 — Supporting Info Sequence conservation (0.22 MB PDF) [file pone.0005410.s003.pdf]

The related C-terminal halves of the MSP3-family of proteins in *P. falciparum* are highly conserved across different geographical isolates.

Genomic DNA was isolated from field isolates in different parts of the world: Brazil (B); Comor Islands (C); Dielmo, Senegal, West Africa (D) and Thailand (T) using standard procedure, or commercially available kit (Qiagen).

MSP3-family gene fragments were amplified from these DNA samples by nested PCR primer sets (different from the one used for sequencing). A control (without template DNA) was included for each round of PCR.

DNA was purified by agarose gel electrophoresis, and sequenced using any one of the primer sets used for cloning of the related C-terminal recombinant protein from the MSP3-multigene family (SI, Table B:2). The sequences were aligned using Gene Jockey software (CLUSTALW alignments).

Shown below are alignments of the predicted translation products of the amplified DNA fragment for each gene. It is clear that the members of the MSP3-family of proteins are very highly conserved (even at the nucleotide level, data not shown).

MSP3.1 C-term amino acid alignments :

```
                         10        20        30        40        50        60
                          |         |         |         |         |         |
Contig# 1       ........................................ ........................
M33D7Saa copie  ---------------------------YEKAKNAYQKANQAVLKAKEASSYDYILGWEFGGGVP
M3B2coraa       -------------------------------------RNQAVLKAKEASSYDYILGWEFGGGVP
M3B3aa copie    SETPEKPSRINLFSRKTKEYAEKAKNAYEKAKNAYQKANQAVLKAKEASSYDYILGWEFGGGVP
M3B4aa copie    ----EKSSKADSISTKTKEYAEKAKNAYEKAKNAYQKANQAVLKAKEASSYDYILGWEFGGGVP
M3B5aa copie    ----EKSSKADSISTKTKEYAEKAKNAYEKAKNAYQKANQAVLKAKEASSYDYILGWEFGGGVP
M3B6aa copie    ----EKPSRINLFSRKTKEYAEKAKNAYEKAKNAYQKANQAVLKAKEASSYDYILGWEFGGGVP
M3SB2aa copie   ---------------------------YEKAKNAYQKANQAVLKAKEASSYDYILGWEFGGGVP
M3SB3aa copie   ---------------------------YEKAKNAYQKANQAVLKAKEASSYDYILGWEFGGGVP
M3SB4aa copie   ---------------------------YEKAKNAYQKANQAVLKAKEASSYDYILGWEFGGGVP
M3SB5aa copie   ---------------------------YEKAKNAYQKANQAVLKAKEASSYDYILGWEFGGGVP
M3D1coraa       ---------------------------------------QAVLKAKEASSYDYILGWEFGGGVP
M3D4coraa       ----------------------------------------AVLKAKEASSYDYILGWEFGGGVP
M3SD2aa copie   ---------------------------YEKAKNAYQKANQAVLKAKEASSYDYILGWEFGGGVP
M3SD3aa copie   ---------------------------YEKAKNAYQKANQAVLKAKEASSYDYILGWEFGGGVP
M3SD4aa copie   ---------------------------YEKAKNAYQKANQAVLKAKEASSYDYILGWEFGGGVP
M3SD5aa copie   ---------------------------YEKAKNAYQKANQAVLKAKEASSYDYILGWEFGGGVP
M3T1coraa       ----------------------------------------AVLKAKEASSYDYILGWEFGGGVP
M3T2aa copie    KDDAEKSSKADSISTKTKEYAEKAKNAYEKAKNAYQKANQAVLKAKEASSYDYILGWEFGGGVP
M3T3coraa       --------------------------------------NQAVLKAKEASSYDYILGWEFGGGVP
M3T5coraa       ----------------------------------------AVLKAKEASSYDYILGWEFGGGVP

                     70        80        90       100       110       120
                      |         |         |         |         |         |
Contig# 1       ............................|...................................
M33D7Saa copie  EHKKEENMLSHLYVSSKDKENISKENDDVLDEKEEEAEETEEEELEEKNEEETESEISEDEEEE
M3B2coraa       EHKKEENMLSHLYVSSKDKENISKENDDVLDEKEEEAEETEEEELEEKNEEETESEISEDEEEE
M3B3aa copie    EHKKEENMLSHLYVSSKDKENISKENDDVLDEKEEEAEETEEEELEEKNEEETESEISEDEEEE
M3B4aa copie    EHKKEENMLSHLYVSSKDKENISKENDDVLDEKEEEAEETEEEELEEKNEEETESEISEDEEEE
M3B5aa copie    EHKKEENMLSHLYVSSKDKENISKENDDVLDEKEEEAEETEEEELEEKNEEETESEISEDEEEE
M3B6aa copie    EHKKEENMLSHLYVSSKDKENISKENDDVLDEKEEEAEETEEEELEEKNEEETESEISEDEEEE
M3SB2aa copie   EHKKEENMLSHLYVSSKDKENISKENDDLLDEKEEEAEETEEEELEEKNEEETESEISEDEEEE
M3SB3aa copie   EHKKEENMLSHLYVSSKDKENISKENDDVLDEKEEEAEETEEEELEEKNEEETESEISEDEEEE
M3SB4aa copie   EHKKEENMLSHLYVSSKDKENISKENDDVLDEKEEEAEETEEEELEEKNEEETESEISEDEEEE
M3SB5aa copie   EHKKEENMLSHLYVSSKDKENISKENDDVLDEKEEEAEETEEEELEEKNEEETESEISEDEEEE
M3D1coraa       EHKKEENMLSHLYVSSKDKENISKENDDVLDEKEEEAEETEEEELEEKNEEETESEISEDEEEE
M3D4coraa       EHKKEENMLSHLYVSSKDKENISKENDDVLDEKEEEAEETEEEELEEKNEEETESEISEDEEEE
M3SD2aa copie   EHKKEENMLSHLYVSSKDKENISKENDDVLDEKEEEAEETEEEELEEKNEEETESEISEDEEEE
M3SD3aa copie   EHKKEENMLSHLYVSSKDKENISKENDDVLDEKEEEAEETEEEELEEKNEEETESEISEDEEEE
M3SD4aa copie   EHKKEENMLSHLYVSSKDKENISKENDDVLDEKEEEAEETEEEELEEKNEEETESEISEDEEEE
M3SD5aa copie   EHKKEENMLSHLYVSSKDKENISKENDDVLDEKEEEAEETEEEELEEKNEEETESEISEDEEEE
M3T1coraa       EHKKEENMLSHLYVSSKDKENISKENDDVLDEKEEEAEETEEEELEEKNEEETESEISEDEEEE
M3T2aa copie    EHKKEENMLSHLYVSSKDKENISKENDDVLDEKEEEAEETEEEELEEKNEEETESEISEDEEEE
M3T3coraa       EHKKEENMLSHLYVSSKDKENISKENDDVLDEKEEEAEETEEEELEEKNEEETESEISEDEEEE
M3T5coraa       EHKKEENMLSHLYVSSKDKENISKENDDVLDEKEEEAEETEEEELEEKNEEETESEISEDEEEE
```

```
                 130       140       150       160       170       180       190
                   |         |         |         |         |         |         |
Contig# 1       •••           ••• ••••|•••••••••••••••••••••••••••••••••••••••••
M33D7Saa copie  EEEEE-KEEENDKKKEQEKEQSNENNDQKKDMEAQNLISKNQNNNEKNVKEAAESIMKTLAGLI
M3B2coraa       EEEEE-KEEENDKKKEQEKEQSNENNDQKKDMEAQNLISKNQNNNEKNVKEAAESIMKTLAGLI
M3B3aa copie    EEE-EEKEEENDKKKEQEKEQSNENNDQKKDMEAQNLISKNQNNNEKNVKEAAESIMKTLAGLI
M3B4aa copie    EEEKEEE-------KEQAKEQSNENNDQKKDMEAQNLISKNQNNNEKNVKEAAESIMKTLAGLI
M3B5aa copie    EEEKEEE-------KEQAKEQSNENNDQKKDMEAQNLISKNQNNNEKNVKEAAESIMKTLAGLI
M3B6aa copie    EEEKEEE-------KEQAKEQSNENNDQKKDMEAQNLISKNQNNNEKNVKEAAESIMKTLAGLI
M3SB2aa copie   EEEKEEE-------KEQAKEQSNENNDQKKDMEAQNLISKNQNNNEKNVKEAAESIMKTLAGLI
M3SB3aa copie   EEEKEEE-------KEQAKEQSNENNDQKKDMEAQNLISKNQNNNEKNVKEAAESIMKTLAGLI
M3SB4aa copie   EEEKEEE-------KEQAKEQSNENNDQKKDMEAQNLISKNQNNNEKNVKEAAESIMKTLAGLI
M3SB5aa copie   EEEKEEE-------KEQAKEQSNENNDQKKDMEAQNLISKNQNNNEKNVKEAAESIMKTLAGLI
M3D1coraa       EEEEE-KEEENDKKKEQEKEQSNENNDQKKDMEAQNLISKNQNNNEKNVKEAAESIMKTLAGLI
M3D4coraa       EEEEEEKEEENDKKKEQEKEQSNENNDQKKDMEAQNLISKNQNNNEKNVKEAAESIMKTLAGLI
M3SD2aa copie   EEEE--KEEENDKKKEQEKEQSNENNDQKKDMEAQNLISKNQNNNEKNVKEAAESIMKTLAGLI
M3SD3aa copie   EEEEE-KEEENDKKKEQEKEQSNENNDQKKDMEAQNLISKNQNNNEKNVKEAAESIMKTLAGLI
M3SD4aa copie   EEEEEEREEENDKKKEQEKEQSSENNDQKKDMEAQNLISKNQNNNEKNVKEAAESIMKTLAGLI
M3SD5aa copie   EEEEEEKEEENDKKKEQEKEQSNENNDQKKDMEAQNLISKNQNNNEKNVKEAAESIMKTLAGLI
M3T1coraa       EEEE--KEEENEKKKEQEKEQSNENNDQKKDMEAQNLISKNQNNNEKNVKEAAESIMKTLAGLI
M3T2aa copie    EEE-EEKEEENDKKKEQEKEQSNENNDQKKDMEAQNLISKNQNNNEKNVKEAAESIMKTLAGLI
M3T3coraa       EEEE--KEEENEKKKEQEKEQSNENNDQKKDMEAQNLISKNQNNNEKNVKEAAESIMKTLAGLI
M3T5coraa       EEEE--KEEENEKKKEQEKEQSNENNDQKKDMEAQNLISKNQNNNEKNVKEAAESIMKTLAGLI

                          200       210
                            |         |
Contig# 1        •••••••••••••••  |
M33D7Saa copie  KGNNQIDSTLKDLVEELSKYFKNH
M3B2coraa       KGNNQIDSTLKDLVEEYP
M3B3aa copie    KGNNQIDST
M3B4aa copie    KGNNQIDSTLKDLVEELSK
M3B5aa copie    KGNNQIDSTLKDLVEEYP
M3B6aa copie    KGNNQIDSTLKDLVEEYP-----N
M3SB2aa copie   KGNNQIDSTLKDLVEELSKYFKNH
M3SB3aa copie   KGNNQIDSTLKDLVEELSKYFKNH
M3SB4aa copie   KGNNQIDSTLKDLVEELSKYFKNH
M3SB5aa copie   MGNNQIDSTLKDLVEELSQYFKNH
M3D1coraa       KGNNQIDSTLKDLVEELSKF--KS
M3D4coraa       KGNNQIDSTLKDLVEELSKY
M3SD2aa copie   KGNNQIDSTLKDLVEELSKYFKNH
M3SD3aa copie   KGNNQIDSTLKDLVEELSKYFKNH
M3SD4aa copie   KGNNQIDSTLKDLVEELSKYFKNH
M3SD5aa copie   KGNNQIDSTLKDLVEELSKYFKNH
M3T1coraa       KGNNQIDSTLKDL
M3T2aa copie    KGNNQIDST
M3T3coraa       KGNNQIDSTLKDLVEEYPN-IKTN
M3T5coraa       KGNNQIDSTLKDLVEEYPN-LNL
```

MSP3.2 C-term amino acid alignments:

```
                   10        20        30        40        50        60
                    |         |         |         |         |         |
Contig# 1    •••••••|  •|•••••••••••••••••••••••••••••••••••••••••••••••••••••••  ••
MSP3.2 3D7   ETNKNPTSHSNSTTTSLNNNILGWEFGGGAPQNGAAEDKKTEYLLEQIKIPSWDRNNIPDENEQVIED
MSP3.2 B2    ETNKNPTPGSKSTTTSLNNNILGWEFGGGAPQNGAAEDKKTEYLLEQIKIPSWDRNNIPDENEQVKED
MSP3.2 B3    ETNKNPTPGSKSTTTSLNNNILGWEFGGGAPQNGAAEDKKTEYLLEQIKIPSWDRNNIPDENEQVKED
MSP3.2 B4    ETNKNPTSHSNSTTTSLNNNILGWEFGGGAPQNGAAEDKKTEYLLEQIKIPSWDRNNIPDENEQVKED
MSP3.2 B5    ETNKNPTSHSNSTTTSLNNNILGWEFGGGAPQNGAAEDKKTEYLLEQIKIPSWDRNNIPDENEQVKED
MSP3.2 B6    ETNKNPTSHSNSTTTSLNNNILGWEFGGGAPQNGAAEDKKTEYLLEQIKIPSWDRNNIPDENEQVKED
MSP3.2 B8    ETNKNPTSHSNSTTTSLNNNILGWEFGGGAPQNGAAEDKKTEYLLEQIKIPSWDRNNIPDENEQVKED
MSP3.2 B9    ETNKNPTPGSKSTTTSLNNNILGWEFGGGAPQNGAAEDKKTEYLLEQIKIPSWDRNNIPDENEQVKED
MSP3.2 B10   ETNKNPTSHSNSTTTSLNNNILGWEFGGGAPQNGAAEDKKTEYLLEQIKIPSWDRNNIPDENEQVKED
MSP3.2 B11   ETNKNPTPGSKSTTTSLNNNILGWEFGGGAPQNGAAEDKKTEYLLEQIKIPSWDRNNIPDENEQVKED
MSP3.2 B12   ETNKNPTPGSKSTTTSLNNNILGWEFGGGAPQNGAAEDKKTEYLLEQIKIPSWDRNNIPDENEQVKED
MSP3.2 C1    ETNKNPTPGSKSTTTSLNNNILGWEFGGGAPQNGAAEDKKTEYLLEQIKIPSWDRNNIPDENEQVKED
MSP3.2 C2    ETNKNPTSHSNSTTTSLNNNILGWEFGGGAPQNGAAEDKKTEYLLEQIKIPSWDRNNIPDENEQVKED
MSP3.2 C3    ETNKNPTSHSNSTTTSLNNNILGWEFGGGAPQNGAAEDKKTEYLLEQIKIPSWDRNNIPDENEQVIED
MSP3.2 C4    ETNKNPTPGSKSTTTSLNNNILGWEFGGGAPQNGAAEDKKTEYLLEQIKIPSWDRNNIPDENEQVKED
MSP3.2 C5    ETNKNPTPGSKSTTTSLNNNILGWEFGGGAPQNGAAEDKKTEYLLEQIKIPSWDRNNIPDENEQVKED
MSP3.2 C6    ETNKNPTSHSNSTTTSLNNNILGWEFGGGAPQNGAAEDKKTEYLLEQIKIPSWDRNNIPDENEQVKED
MSP3.2 C7    ETNKNPTPGSKSTTTSLNNNILGWEFGGGAPQNGAAEDKKTEYLLEQIKIPSWDRNNIPDENEQVKED
MSP3.2 C9    ETNKNPTPGSKSTTTSLNNNILGWEFGGGAPQNGAAEDKKTEYLLEQIKIPSWDRNNIPDENEQVKED
MSP3.2 C10   ETNKNPTSHSNSTTTSLNNNILGWEFGGGAPQNGAAEDKKTEYLLEQIKIPSWDRNNIPDENEQVKED
MSP3.2 C11   ETNKNPTSHSNSTTTSLNNNILGWEFGGGAPQNGAAEDKKTEYLLEQIKIPSWDRNNIPDENEQVIED
MSP3.2 C12   ETNKNPTSHSNSTTTSLNNNILGWEFGGGAPQNGAAEDKKTEYLLEQIKIPSWDRNNIPDENEQVIED
MSP3.2 D1    ETNKNPTSHSNSTTTSLNNNILGWEFGGGAPQNGAAEDKKTEYLLEQIKIPSWDRNNIPDENEQVKED
MSP3.2 D2    ETNKNPTPGSKSTTTSLNNNILGWEFGGGAPQNGAAEDKKTEYLLEQIKIPSWDRNNIPDENEQVIED
MSP3.2 D3    ETNKNPTSHSNSTTTSLNNNILGWEFGGGAPQNGAAEDKKTEYLLEQIKIPSWDRNNIPDENEQVKED
MSP3.2 D4    ETNKNPTPGSKSTTTSLNNNILGWEFGGGAPQNGAAEDKKTEYLLEQIKIPSWDRNNIPDENEQVKED
MSP3.2 D6    ETNKNPTSHSNSTTTSLNNNILGWEFGGGAPQNGAAEDKKTEYLLEQIKIPSWDRNNIPDENEQVIED
MSP3.2 D7    ETNKNPTSHSNSTTTSLNNNILGWEFGGGAPQNGAAEDKKTEYLLEQIKIPSWDRNNIPDENEQVKED
MSP3.2 D8    ETNKNPTSHSNSTTTSLNNNILGWEFGGGAPQNGAAEDKKTEYLLEQIKIPSWDRNNIPDENEQVKED
MSP3.2 D9    ETNKNPTSHSNSTTTSLNNNILGWEFGGGAPQNGAAEDKKTEYLLEQIKIPSWDRNNIPDENEQVIED
MSP3.2 D11   ETNKNPTPGSKSTTTSLNNNILGWEFGGGAPQNGAAEDKKTEYLLEQIKIPSWDRNNIPDENEQVKED
MSP3.2 D13   ETNKNPTPGSKSTTTSLNNNILGWEFGGGAPQNGAAEDKKTEYLLEQIKIPSWDRNNIPDENEQVKED
MSP3.2 T1    ETNKNPTPGSKSTTTSLNNNILGWEFGGGAPQNGAAEDKKTEYLLEQIKIPSWDRNNIPDENEQVKED
MSP3.2 T2    ETNKNPTPGSKSTTTSLNNNILGWEFGGGAPQNGAAEDKKTEYLLEQIKIPSWDRNNIPDENEQVKED
MSP3.2 T32   ETNKNPTPGSKSTTTSLNNNILGWEFGGGAPQNGAAEDKKTEYLLEQIKIPSWDRNNIPDENEQVKED
MSP3.2 T4    ETNKNPTPGSKSTTTSLNNNILGWEFGGGAPQNGAAEDKKTEYLLEQIKIPSWDRNNIPDENEQVKED
MSP3.2 T5    ETNKNPTSHSNSTTTSLNNNILGWEFGGGAPQNGAAEDKKTEYLLEQIKIPSWDRNNIPDENEQVIED
```

```
              70        80        90        100       110       120       130
               |         |         |         |         |         |         |
Contig# 1    ............  ...............................|.................
MSP3.2 3D7   PQEDNKDEDEDE--ETETENLETEDDNNEEIEENEEDDIDEESVEEKEEEEEKKEEEEKKEEKKEEKK
MSP3.2 B2    PQEDNKDEDEDE--ETETENLETEDDNNEEIEENEEDDIDEESVEEKEEEEEKKEEEEKKEEKKEEKK
MSP3.2 B3    PQEDNKDEDEDE--ETETENLETEDDNNEEIEENEEDDIDEESVEEKEEEEEKKEEEEKKEEKKEEKK
MSP3.2 B4    PQEDNKDEDEDE--ETETENLETEDDNNEEIEENEEDDIDEESVEEKEEEEEKKEEEEKKEEKKEEKK
MSP3.2 B5    PQEDNKDEDEDE--ETETENLETEDDNNEEIEENEEDDIDEESVEEKEEEEEKKEEEEKKEEKKEEKK
MSP3.2 B6    PQEDNKDEDEDE--ETETENLETEDDNNEEIEENEEDDIDEESVEEKEEEEEKKEEEEKKEEKKEEKK
MSP3.2 B8    PQEDNKDEDEDE--ETETENLETEDDNNEEIEENEEDDIDEESVEEKEEEEEKKEEEEKKEEKKEEKK
MSP3.2 B9    PQEDNKDEDEDE--ETETENLETEDDNNEEIEENEEDDIDEESVEEKEEEEEKKEEEEKKEEKKEEKK
MSP3.2 B10   PQEDNKDEDEDE--ETETENLETEDDNNEEIEENEEDDIDEESVEEKEEEEEKKEEEEKKEEKKEEKK
MSP3.2 B11   PQEDNKDEDEDE--ETETENLETEDDNNEEIEENEEDDIDEESVEEKEEEEEKKEEEEKKEEKKEEKK
MSP3.2 B12   PQEDNKDEDEDE--ETETENLETEDDNNEEIEENEEDDIDEESVEEKEEEEEKKEEEEKKEEKKEEKK
MSP3.2 C1    PQEDNKDEDEDE--ETETENLETEDDNNEEIEENEEDDIDEESVEEKEEEEEKKEEEEKKEEKKEEKK
MSP3.2 C2    PQEDNKDEDEDEDEETETENLETEDDNNEEIEENEEDDIDEESVEEKEEEEEKKEEEEKKEEKKEEKK
MSP3.2 C3    PQEDNKDEDEDE--ETETENLETEDDNNEEIEENEEDDIDEESVEEKEEEEEKKEEEEKKEEKKEEKK
MSP3.2 C4    PQEDNKDEDEDE--ETETENLETEDDNNEEIEENEEDDIDEESVEEKEEEEEKKEEEEKKEEKKEEKK
MSP3.2 C5    PQEDNKDEDEDE--ETETENLETEDDNNEEIEENEEDDIDEESVEEKEEEEEKKEEEEKKEEKKEEKK
MSP3.2 C6    PQEDNKDEDEDE--ETETENLETEDDNNEEIEENEEDDIDEESVEEKEEEEEKKEEEEKKEEKKEEKK
MSP3.2 C7    PQEDNKDEDEDE--ETETENLETEDDNNEEIEENEEDDIDEESVEEKEEEEEKKEEEEKKEEKKEEKK
MSP3.2 C9    PQEDNKDEDEDE--ETETENLETEDDNNEEIEENEEDDIDEESVEEKEEEEEKKEEEEKKEEKKEEKK
MSP3.2 C10   PQEDNKDEDEDE--ETETENLETEDDNNEEIEENEEDDIDEESVEEKEEEEEKKEEEEKKEEKKEEKK
MSP3.2 C11   PQEDNKDEDEDE--ETETENLETEDDNNEEIEENEEDDIDEESVEEKEEEEEKKEEEEKKEEKKEEKK
MSP3.2 C12   PQEDNKDEDEDE--ETETENLETEDDNNEEIEENEEDDIDEESVEEKEEEEEKKEEEEKKEEKKEEKK
MSP3.2 D1    PQEDNKDEDEDE--ETETENLETEDDNNEEIEENEEDDIDEESVEEKEEEEEKKEEEEKKEEKKEEKK
MSP3.2 D2    PQEDNKDEDEDE--ETETENLETEDDNNEEIEENEEDDIDEESVEEKEEEEEKKEEEEKKEEKKEEKK
MSP3.2 D3    PQEDNKDEDEDE--ETETENLETEDDNNEEIEENEEDDIDEESVEEKEEEEEKKEEEEKKEEKKEEKK
MSP3.2 D4    PQEDNKDEDEDEDEETETENLETEDDNNEEIEENEEDDIDEESVEEKEEEEEKKEEEEKKEEKKEEKK
MSP3.2 D6    PQEDNKDEDEDE--ETETENLETEDDNNEEIEENEEDDIDEESVEEKEEEEEKKEEEEKKEEKKEEKK
MSP3.2 D7    PQEDNKDEDEDE--ETETENLETEDDNNEEIEENEEDDIDEESVEEKEEEEEKKEEEEKKEEKKEEKK
MSP3.2 D8    PQEDNKDEDEDE--ETETENLETEDDNNEEIEENEEDDIDEESVEEKEEEEEKKEEEEKKEEKKEEKK
MSP3.2 D9    PQEDNKDEDEDE--ETETENLETEDDNNEEIEENEEDDIDEESVEEKEEEEEKKEEEEKKEEKKEEKK
MSP3.2 D11   PQEDNKDEDEDEDEETETENLETEDDNNEEIEENEEDDIDEESVEEKEEEEEKKEEEEKKEEKKEEKK
MSP3.2 D13   PQEDNKDEDEDEDEETETENLETEDDNNEEIEENEEDDIDEESVEEKEEEEEKKEEEEKKEEKKEEKK
MSP3.2 T1    PQEDNKDEDEDE  ETETENLETEDDNNEEIEENEEDDIDEESVEEKEEEEEKKEEEEKKEEKKEEKK
MSP3.2 T2    PQEDNKDEDEDE--ETETENLETEDDNNEEIEENEEDDIDEESVEEKEEEEEKKEEEEKKEEKKEEKK
MSP3.2 T32   PQEDNKDEDEDE--ETETENLETEDDNNEEIEENEEDDIDEESVEEKEEDEEKKEEEEKKEEKKEEKK
MSP3.2 T4    PQEDNKDEDEDE--ETETENLETEDDNNEEIEENEEDDIDEESVEEKEEEEEKKEEEEKKEEKKEEKK
MSP3.2 T5    PQEDNKDEDEDE--ETETENLETEDDNNEEIEENEEDDIDEESVEEKEEEEEKKEEEEKKEEKKEEKK
```

```
                 140       150       160       170       180       190
                   |         |         |         |         |         |
Contig# 1         .......................................|..................
MSP3.2 3D7      PDNEITNEVKEEQKYSSPSDINAQNLISNKNKKNDETKKTAENIVKTLVGLFNEKNEIDSTIN
MSP3.2 B2       PDNEITNEVKEEQKYSSPSDINAQNLISNKNKKNDETKKTAENIVKTLVGLFNEKNEIDSTIN
MSP3.2 B3       PDNEITNEVKEEQKYSSPSDINAQNLISNKNKKNDETKKTAENIVKTLVGLFNEKNEIDSTIN
MSP3.2 B4       PDNEITNEVKEEQKYSSPSDINAQNLISNKNKKNDETKKTAENIVKTLVGLFNEKNEIDSTIN
MSP3.2 B5       PDNEITNEVKEEQKYSSPSDINAQNLISNKNKKNDETKKTAENIVKTLVGLFNEKNEIDSTIN
MSP3.2 B6       PDNEITNEVKEEQKYSSPSDINAQNLISNKNKKNDETKKTAENIVKTLVGLFNEKNEIDSTIN
MSP3.2 B8       PDNEITNEVKEEQKYSSPSDINAQNLISNKNKKNDETKKTAENIVKTLVGLFNEKNEIDSTIN
MSP3.2 B9       PDNEITNEVKEEQKYSSPSDINAQNLISNKNKKNDETKKTAENIVKTLVGLFNEKNEIDSTIN
MSP3.2 B10      PDNEITNEVKEEQKYSSPSDINAQNLISNKNKKNDETKKTAENIVKTLVGLFNEKNEIDSTIN
MSP3.2 B11      PDNEITNEVKEEQKYSSPSDINAQNLISNKNKKNDETKKTAENIVKTLVGLFNEKNEIDSTIN
MSP3.2 B12      PDNEITNEVKEEQKYSSPSDINAQNLISNKNKKNDETKKTAENIVKTLVGLFNEKNEIDSTIN
MSP3.2 C1       PDNEITNEVKEEQKYSSPSDINAQNLISNKNKKNDETKKTAENIVKTLVGLFNEKNEIDSTIN
MSP3.2 C2       LDNEITNEVKEEQKYSSPSDINAQNLISNKNKKNDETKKTAENMVKTLVGLFNEKNEIDSTIN
MSP3.2 C3       PDNEITNEVKEEQKYSSPSDINAQNLISNKNKKNDETKKTAENIVKTLVGLFNEKNEIDSTIN
MSP3.2 C4       PDNEITNEVKEEQKYSSPSDINAQNLISNKNKKNDETKKTAENIVKTLVGLFNEKNEIDSTIN
MSP3.2 C5       PDNEITNEVKEEQKYSSPSDINAQNLISNKNKKNDETKKTAENIVKTLVGLFNEKNEIDSTIN
MSP3.2 C6       PDNEITNEVKEEQKYSSPSDINAQNLISNKNKKNDETKKTAENIVKTLVGLFNEKNEIDSTIN
MSP3.2 C7       PDNEITNEVKEEQKYSSPSDINAQNLISNKNKKNDETKKTAENIVKTLVGLFNEKNEIDSTIN
MSP3.2 C9       PDNEITNEVKEEQKYSSPSDINAQNLISNKNKKNDETKKTAENIVKTLVGLFNEKNEIDSTIN
MSP3.2 C10      PDNEITNEVKEEQKYSSPSDINAQNLISNKNKKNDETKKTAENIVKTLVGLFNEKNEIDSTIN
MSP3.2 C11      PDNEITNEVKEEQKYSSPSDINAQNLISNKNKKNDETKKTAENIVKTLVGLFNEKNEIDSTIN
MSP3.2 C12      PDNEITNEVKEEQKYSSPSDINAQNLISNKNKKNDETKKTAENIVKTLVGLFNEKNEIDSTIN
MSP3.2 D1       PDNEITNEVKEEQKYSSPSDINAQNLISNKNKKNDETKKTAENIVKTLVGLFNEKNE
MSP3.2 D2       PDNEITNEVKEEQKYSSPSDINAQNLISNKNKKNDETKKTAENIVKTLVGLFNEKNEIDSTIN
MSP3.2 D3       PDNEITNEVKEEQKYSSPSDINAQNLISNKNKKNDETKKTAENIVKTLVGLFNEKNEIDSTIN
MSP3.2 D4       PDNEITNEVKEEQKYSSPSDINAQNLISNKNKKNDETKKTAENIVKTLVGLFNEKNEIDSTIN
MSP3.2 D6       PDNEITNEVKEEQKYSSPSDINAQNLISNKNKKNDETKKTAENIVKTLVGLFNEKNEIDSTIN
MSP3.2 D7       PDNEITNEVKEEQKYSSPSDINAQNLISNKNKKNDETKKTAENIVKTLVGLFNEKNEIDSTIN
MSP3.2 D8       PDNEITNEVKEEQKYSSPSDINAQNLISNKNKKNDETKKTAENIVKTLVGLFNEKNEIDSTIN
MSP3.2 D9       PDNEITNEVKEEQKYSSPSDINAQNLISNKNKKNDETKKTAENIVKTLVGLFNEKNEIDSTIN
MSP3.2 D11      PDNEITNEVKEEQKYSSPSDINAQNLISNKNKKNDETKKTAENIVKTLVGLFNEKNEIDSTIN
MSP3.2 D13      PDNEITNEVKEEQKYSSPSDINAQNLISNKNKKNDETKKTAENIVKTLVGLFNEKNEIDSTIN
MSP3.2 T1       PDNEITNEVKEEQKYSSPSDINAQNLISNKNKKNDETKKTAENIVKTLVGLFNEK
MSP3.2 T2       PDNEITNEVKEEQKYSSPSDINAQNLISNKNKKNDETKKTAENIVKTLVGLFNEKNEIDSTIN
MSP3.2 T32      PDNEITNEVKEEQKYSSPSDINAQNLISNKNKKNDETKKTAENIVKTLVGLFNEKNEIDSTIN
MSP3.2 T4       PDNEITNEVKEEQKYSSPSDINAQNLISNKNKKNDETKKTAENIVKTLVGLFNEKNEIDSTIN
MSP3.2 T5       PDNEITNEVKEEQKYSSPSDINAQNLISNKNKKNDETKKTAENIVKTLVGLFNEKNEIDSTIN
```

MSP3.3 C-term amino acid alignments:

```
                         10        20        30        40        50        60
                          |         |         |         |         |         |
Contig# 1                 .................................................. .......
MSP3.3 3D7  VDSKTKSNEKGRPPTYSPILDDGIEFSGGLYFNEKKSTEENKQKNVLESVNLTSWDKEDIVKENEDVK
MSP3.3 B3   VDSKTKSNEKGRPPTYSPILDDGIEFSGGLYFNEKKSTEENKQKNVLESVNLTSWDKEDIVKENEDVK
MSP3.3 B4   VDSKTKSNEKGRPPTYSPILDDGIEFSGGLYFNEKKSTEENKQKNVLESVNLTSWDKEDIVKENEDVK
MSP3.3 B5   VDSKTKSNEKGRPPTYSPILDDGIEFSGGLYFNEKKSTEENKQKNVLESVNLTSWDKEDIVKENEDVK
MSP3.3 B8   VDSKTKSNEKGRPPTYSPILDDGIEFSGGLYFNEKKSTEENKQKNVLESVNLTSWDKEDIVKENEDVK
MSP3.3 B9   VDSKTKSNEKGRPPTYSPILDDGIEFSGGLYFNEKKSTEENKQKNVLESVNLTSWDKEDIVKENEDVK
MSP3.3 B10  VDSKTKSNEKGRPPTYSPILDDGIEFSGGLYFNEKKSTEENKQKNVLESVNLTSWDKEDIVKENEDVK
MSP3.3 B11  VDSKTKSNEKGRPPTYSPILDDGIEFSGGLYFNEKKSTEENKQKNVLESVNLTSWDKEDIVKENEDVK
MSP3.3 B12  VDSKTKSNEKGRPPTYSPILDDGIEFSGGLYFNEKKSTEENKQKNVLESVNLTSWDKEDIVKENEDVK
MSP3.3 B2   VDSKTKSNEKGRPPTYSPILDDGIEFSGGLYFNEKKSTEENKQKNVLESVNLTSWDKEDIVKENEDVK
MSP3.3 B5   VDSKTKSNEKGRPPTYSPILDDGIEFSGGLYFNEKKSTEENKQKNVLESVNLTSWDKEDIVKENEDVK
MSP3.3 C1   VDSKTKSNEKGRPPTYSPILDDGIEFSGGLYFNEKKSTEENKQKNVLESVNLTSWDKEDIVKENEDVK
MSP3.3 C2   VDSKTKSNEKGRPPTYSPILDDGIEFSGGLYFNEKKSTEENKQKNVLESVNLTSWDKEDIVKENEDVK
MSP3.3 C3   VDSKTKSNEKGRPPTYSPILDDGIEFSGGLYFNEKKSTEENKQKNVLESVNLTSWDKEDIVKENEDVK
MSP3.3 C4   VDSKTKSNEKGRPPTYSPILDDGIEFSGGLYFNEKKSTEENKQKNVLESVNLTSWDKEDIVKENEDVK
MSP3.3 C5   VDSKTKSNEKGRPPTYSPILDDGIEFSGGLYFNEKKSTEENKQKNVLESVNLTSWDKEDIVKENEDVK
MSP3.3 C6   VDSKTKSNEKGRPPTYSPILDDGIEFSGGLYFNEKKSTEENKQKNVLESVNLTSWDKEDIVKENEDVK
MSP3.3 C7   VDSKTKSNEKGRPPTYSPILDDGIEFSGGLYFNEKKSTEENKQKNVLESVNLTSWDKEDIVKENEDVK
MSP3.3 C9   VDSKTKSNEKGRPPTYSPILDDGIEFSGGLYFNEKKSTEENKQKNVLESVNLTSWDKEDIVKENEDVK
MSP3.3 C10  VDSKTKSNEKGRPPTYSPILDDGIEFSGGLYFNEKKSTEENKQKNVLESVNLTSWDKEDIVKENEDVK
MSP3.3 C11  VDSKTKSNEKGRPPTYSPILDDGIEFSGGLYFNEKKSTEENKQKNVLESVNLTSWDKEDIVKENEDVK
MSP3.3 D1   VDSKTKSNEKGRPPTYSPILDDGIEFSGGLYFNEKKSTEENKQKNVLESVNLTSWDKEDIVKENEDVK
MSP3.3 D4   VDSKTKSNEKGRPPTYSPILDDGIEFSGGLYFNEKKSTEENKQKNVLESVNLTSWDKEDIVKENEDVK
MSP3.3 D5   VDSKTKSNEKGRPPTYSPILDDGIEFSGGLYFNEKKSTEENKQKNVLESVNLTSWDKEDIVKENEDVK
MSP3.3 D6   ---KNKSNEKGRPPTYSPILDDGIEFSGGLYFNEKKSTEENKQKNVLESVNLTSWDKEDIVKENEDVK
MSP3.3 D7   ----TKSNEKGRPPTYSPILDDGIEFSGGLYFNEKKSTEENKQKNVLESVNLTSWDKEDIVKENEDVK
MSP3.3 D8   ----TKSNEKGRPPTYSPILDDGIEFSGGLYFNEKKSTEENKQKNVLESVNLTSWDKEDIVKENEDVK
MSP3.3 D9   --------EEGRPPTYSPILDDGIEFSGGLYFNEKKSTEENKQKNVLESVNLTSWDKEDIVKENEDVK
MSP3.3 D10  --------EKGRPPTYSPILDDGIEFSGGLYFNEKKSTEENKQKNVLESVNLTSWDKEDIVKENEDVK
MSP3.3 D11  --     SEKGRPPTYSPILDDGIEFSGGLYFNEKKSTEENKQKNVLESVNLTSWDKEDIVKENEDVK
MSP3.3 D12   --KTKSNEKGRPPTYSPILDDGIEFSGGLYFNEKKSTEENKQKNVLESVNLTSWDKEDIVKENEDVK
MSP3.3 D13  ----------GRPPTYSPILDDGIEFSGGLYFNEKKSTEENKQKNVLESVNLTSWDKEDIDKENEDVK
MSP3.3 T1   VDSKTKSNEKGRPPTYSPILDDGIEFSGGLYFNEKKSTEENKQKNVLESVNLTSWDKEDIVKENEDVK
MSP3.3 T2   VDSKTKSNEKGRPPTYSPILDDGIEFSGGLYFNEKKSTEENKQKNVLESVNLTSWDKEDIVKENEDVK
MSP3.3 T3   VDSKTKSNEKGRPPTYSPILDDGIEFSGGLYFNEKKSTEENKQKNVLESVNLTSWDKEDIVKENEDVK
MSP3.3 T4   VDSKTKSNEKGRPPTYSPILDDGIEFSGGLYFNEKKSTEENKQKNVLESVNLTSWDKEDIVKENEDVK
MSP3.3 T5   VDSKTKSNEKGRPPTYSPILDDGIEFSGGLYFNEKKSTEENKQKNVLESVNLTSWDKEDIVKENEDVK
```

```
            70        80        90       100       110       120       130
             |         |         |         |         |         |         |
Contig# 1   .........................................|..........................
MSP3.3 3D7  DEKDEDDEEEEEKYENEIIKQPEDILDEEEVLEEEILEENKNDTVDTSDLEKKNIPDLSNDNNYYSLI
MSP3.3 B3   DEKDEDDEEEEEKYENEIIKQPEDILDEEEVLEEEILEENKNDTVDTSDLEKKNIPDLSNDNNYYSLI
MSP3.3 B4   DEKDEDDEEEEEKYENEIIKQPEDILDEEEVLEEEILEENKNDTVDTSDLEKKNIPDLSNDNNYYSLI
MSP3.3 B5   DEKDEDDEEEEEKYENEIIKQPEDILDEEEVLEEEILEENKNDTVDTSDLEKKNIPDLSNDNNYYSLI
MSP3.3 B8   DEKDEDDEEEEEKYENEIIKQPEDILDEEEVLEEEILEENKNDTVDTSDLEKKNIPDLSNDNNYYSLI
MSP3.3 B9   DEKDEDDEEEEEKYENEIIKQPEDILDEEEVLEEEILEENKNDTVDTSDLEKKNIPDLSNDNNYYSLI
MSP3.3 B10  DEKDEDDEEEEEKYENEIIKQPEDILDEEEVLEEEILEENKNDTVDTSDLEKKNIPDLSNDNNYYSLI
MSP3.3 B11  DEKDEDDEEEEEKYENEIIKQPEDILDEEEVLEEEILEENKNDTVDTSDLEKKNIPDLSNDNNYYSLI
MSP3.3 B12  DEKDEDDEEEEEKYENEIIKQPEDILDEEEVLEEEILEENKNDTVDTSDLEKKNIPDLSNDNNYYSLI
MSP3.3 B2   DEKDEDDEEEEEKYENEIIKQPEDILDEEEVLEEEILEENKNDTVDTSDLEKKNIPDLSNDNNYYSLI
MSP3.3 B5   DEKDEDDEEEEEKYENEIIKQPEDILDEEEVLEEEILEENKNDTVDTSDLEKKNIPDLSNDNNYYSLI
MSP3.3 C1   DEKDEDDEEEEEKYENEIIKQPEDILDEEEVLEEEILEENKNDTVDTSDLEKKNIPDLSNDNNYYSLI
MSP3.3 C2   DEKDEDDEEEEEKYENEIIKQPEDILDEEEVLEEEILEENKNDTVDTSDLEKKNIPDLSNDNNYYSLI
MSP3.3 C3   DEKDEDDEEEEEKYENEIIKQPEDILDEEEVLEEEILEENKNDTVDTSDLEKKNIPDLSNDNNYYSLI
MSP3.3 C4   DEKDEDDEEEEEKYENEIIKQPEDILDEEEVLEEEILEENKNDTVDTSDLEKKNIPDLSNDNNYYSLI
MSP3.3 C5   DEKDEDDEEEEEKYENEIIKQPEDILDEEEVLEEEILEENKNDTVDTSDLEKKNIPDLSNDNNYYSLI
MSP3.3 C6   DEKDEDDEEEEEKYENEIIKQPEDILDEEEVLEEEILEENKNDTVDTSDLEKKNIPDLSNDNNYYSLI
MSP3.3 C7   DEKDEDDEEEEEKYENEIIKQPEDILDEEEVLEEEILEENKKDTVDTSDLEKKNIPDLSNDNNYYSLI
MSP3.3 C9   DEKDEDDEEEEEKYENEIIKQPEDILDEEEVLEEEILEENKNDTVDTSDLEKKNIPDLSNDNNYYSLI
MSP3.3 C10  DEKDEDDEEEEEKYENEIIKQPEDILDEEEVLEEEILEENKNDTVDTSDLEKKNIPDLSNDNNYYSLI
MSP3.3 C11  DEKDEDDEEEEEKYENEIIKQPEDILDEEEVLEEEILEENKKDTVDTSDLEKKNIPDLSNDNNYYSLI
MSP3.3 D1   DEKDEDDEEEEEKYENEIIKQPEDILDEEEVLEEEILEENKNDTVDTSDLEKKNIPDLSNDNNYYSLI
MSP3.3 D4   DEKDEDDEEEEEKYENEIIKQPEDILDEEEVLEEEILEENKNDTVDTSDLEKKNIPDLSNDNNYYSLI
MSP3.3 D5   DEKDEDDEEEEEKYENEIIKQPEDILDEEEVLEEEILEENKNDTVDTSDLEKKNIPDLSNDNNYYSLI
MSP3.3 D6   DEKDEDDEEEEEKYENEIIKQPEDILDEEEVLEEEILEENKNDTVDTSDLEKKNIPDLSNDNNYYSLI
MSP3.3 D7   DEKDEDDEEEEEKYENEIIKQPEDILDEEEVLEEEILEENKKDTVDTSDLEKKNIPDLSNDNNYYSLI
MSP3.3 D8   DEKDEDDEEEEEKYENEIIKQPEDILDEEEVLEEEILEENKNDTVDTSDLEKKNIPDLSNDNNYYSLI
MSP3.3 D9   DEKDEDDEEEEEKYENEIIKQPEDILDEEEVLEEEILEENKNDTVDTSDLEKKNIPDLSNDNNYYSLI
MSP3.3 D10  DEKDEDDEEEEEKYENEIIKQPEDILDEEEVLEEEILEENKNDTVDTSDLEKKNIPDLSNDNNYYSLI
MSP3.3 D11  DEKDEDDEEEEEKYENEIIKQPEDILDEEEVLEEEILEENKNDTVDTSDLEKKNIPDLSNDNNYYSLI
MSP3.3 D12  DEKDEDDEEEEEKYENEIIKQPEDILDEEEVLEEEILEENKNDTVDTSDLEKKNIPDLSNDNNYYSLI
MSP3.3 D13  DEKDEDDEEEEEKYENEIIKQPEDILDEEEVLEEEILEENKNDTVDTSDLEKKNIPDLSNDNNYYSLI
MSP3.3 T1   DEKDEDDEEEEEKYENEIIKQPEDILDEEEVLEEEILEENKNDTVDTSDLEKKNIPDLSNDNNYYSLI
MSP3.3 T2   DEKDEDDEEEEEKYENEIIKQPEDILDEEEVLEEEILEENKNDTVDTSDLEKKNIPDLSNDNNYYSLI
MSP3.3 T3   DEKDEDDEEEEEKYENEIIKQPEDILDEEEVLEEEILEENKNDTVDTSDLEKKNIPDLSNDNNYYSLI
MSP3.3 T4   DEKDEDDEEEEEKYENEIIKQPEDILDEEEVLEEEILEENKNDTVDTSDLEKKNIPDLSNDNNYYSLI
MSP3.3 T5   DEKDEDDEEEEEKYENEIIKQPEDILDEEEVLEEEILEENKNDTVDTSDLEKKNIPDLSNDNNYYSLI
```

```
                 140       150       160       170       180
                   |         |         |         |         |
Contig# 1    .................................|...............
MSP3.3 3D7   YKNYKDNDKSEKTAQTLITALISLLNGKNELDATIRRLKHRFMEFFTYN
MSP3.3 B3    YKNYKDNDKSEKTAQTLITALISLLNGKNELDATIRRLKHRFMEFFTYN
MSP3.3 B4    YKNYKDNDKSEKTAQTLITALISLLNGK
MSP3.3 B5    YKNYKDNDKSEKTAQTLITALISLLNG
MSP3.3 B8    YKNYKDNDKSEKTAQTLITALISLLNGKNELDATIRRLKHRFMEFFTYN
MSP3.3 B9    YKNYKDNDKSEKTAQTLITALISLLNGKNELDATIRRLKHRFMEFFTYN
MSP3.3 B10   YKNYKDNDKSEKTAQTLITALISLLNGKNELDATIRRLKHRFMEFFTYN
MSP3.3 B11   YKNYKDNDKSEKTAQTLITALISLLNGKNELDATIRRLKHRFMEFFTYN
MSP3.3 B12   YKNYKDNDKSEKTAQTLITALISLLNGKNELDATIRRLKHRFMEFFTYN
MSP3.3 B2    YKNYKDNDKSEKTAQTLITALISLLNGKNELDATIRRLKHRFMEFFTYN
MSP3.3 B5    YKNYKDNDKSEKTAQTLITALISLLNGKNELDATIRRLKHRFMEFFTYN
MSP3.3 C1    YKNYKDNDKSEKTAQTLITALISLLNGKNELDATIRRLKHRFMEFFTYN
MSP3.3 C2    YKNYKDNDKSEKTAQTLITALISLLNGKNELDATIRRLKHRFMEFFTYN
MSP3.3 C3    YKNYKDNDKSEKTAQTLITALISLLNGKNELDATIRRLKHRFMEFFTYN
MSP3.3 C4    YKNYKDNDKSEKTAQTLITALISLLNGKNELDATIRRLKHRFMEFFTYN
MSP3.3 C5    YKNYKDNDKSEKTAQTLITALISLLNGKNELDATIRRLKHRFMEFFTYN
MSP3.3 C6    YKNYKDNDKSEKTAQTLITALISLLNGKNELDATIRRLKHRFMEFFTYN
MSP3.3 C7    YKNYKDNDKSEKTAQTLITALISLLNGKNELDATIRRLKHRFMEFFTYN
MSP3.3 C9    YKNYKDNDKSEKTAQTLITALISLLNGKNELDATIRRLKHRFMEFFTYN
MSP3.3 C10   YKNYKDNDKSEKTAQTLITALISLLNGKNELDATIRRLKHRFMEFFTYN
MSP3.3 C11   YKNYKDNDKSEKTAQTLITALISLLNGKNELDATIRRLKHRFMEFFTYN
MSP3.3 D1    YKNYKDNDKSEKTAQTLITALISLLNGKNELDAAIRR
MSP3.3 D4    YKNYKDNDKSEKTAQTLITALISLLNGKNELDATIRR
MSP3.3 D5    YKNYKDNDKSEKTAQTLITALISLLNGKNELDATIRR
MSP3.3 D6    YKNYKDNDKSEKTAQTLITALISLLNGKNELDATIRRLKHRFMEFFTYN
MSP3.3 D7    YKNYKDNDKSEKTAQTLITALISLLNGKNELDATIRRLKHRFMEFFTYN
MSP3.3 D8    YKNYKDNDKSEKTAQTLITALISLLNGKNELDATIRRLKHRFMEFFTYN
MSP3.3 D9    YKNYKDNDKSEKTAQTLITALISLLNGKNELDATIRRLKHRFMEFFTYN
MSP3.3 D10   YKNYKDNDKSEKTAQTLITALISLLNGKNELDATIRRLKHRFMEFFTYN
MSP3.3 D11   YKNYKDNDKSEKTAQTLITALISLLNGKNELDATIRRLKHRFMEFFTYN
MSP3.3 D12   YKNYKDNDKSEKTAQTLITALISLLNGKNELDATIRRLKHRFMEFFTYN
MSP3.3 D13   YKNYKDNDKSEKTAQTLITALISLLNGKNELDATIRRLKHRFMEFFTYN
MSP3.3 T1    YKNYKDNDKSEKTAQTLITALISLLNGKNE
MSP3.3 T2    YKNYKDNDKSEKTAQTLITALISLLNGKNE
MSP3.3 T3    YKNYKDNDKSEKTAQTLITALISLLNGKNE
MSP3.3 T4    YKNYKDNDKSEKTAQTLITALISLLNGK
MSP3.3 T5    YKNYKDNDKSEKTAQTLITALISLLNGK
```

MSP3.4 C-term amino acid alignments:

```
                       10        20        30        40        50        60
                        |         |         |         |         |         |
Contig# 1                                  .........................................
MSP3.4 3D7   ---------------------------DNVNSVTQRGNNNYNNNLERGLGSGALPGTNIITEEKYSLE
MSP3.4 B2    QRGNITTSQGNSHRATVVQQADQTNRLDNVNSVTQRGNNNYNNNLERGLGSGALPGTNIITEEKYSLE
MSP3.4 B3    QRGNITTSQGNSHRATVVQQADQTNRLDNVNSVTQRGNNNYNNNLERGLGSGALPGTNIITEEKYSLE
MSP3.4 B4    QRGNITTSQGNSHRATVVQQADQTNRLDNVNSVTQRGNNNYNNNLERGLGSGALPGTNIITEEKYSLE
MSP3.4 B5    QRGNITTSQGNSHRATVVQQADQTNRLDNVNSVTQRGNNNYNNNLERGLGSGALPGTNIITEEKYSLE
MSP3.4 B6    QRGNITTSQGNSHRATVVQQADQTNRLDNVNSVTQRGNNNYNNNLERGLGSGALPGTNIITEEKYSLE
MSP3.4 B8    QRGNITTSQGNSHRATVVQQADQTNRLDNVNSVTQRGNNNYNNNLERGLGSGALPGTNIITEEKYSLE
MSP3.4 B9    QRGNITTSQGNSHRATVVQQADQTNRLDNVNSVTQRGNNNYNNNLERGLGSGALPGTNIITEEKYSLE
MSP3.4 B10   QRGNITTSQGNSHRATVVQQADQTNRLDNVNSVTQRGNNNYNNNLERGLGSGALPGTNIITEEKYSLE
MSP3.4 B11   QRGNITTSQGNSHRATVVQQADQTNRLDNVNSVTQRGNNNYNNNLERGLGSGALPGTNIITEEKYSLE
MSP3.4 B12   QRGNITTSQGNSHRATVVQQADQTNRLDNVNSVTQRGNNNYNNNLERGLGSGALPGTNIITEEKYSLE
MSP3.4 C1    QRGNITTSQGNSHRATVVQQADQTNRLDNVNSVTQRGNNNYNNNLERGLGSGALPGTNIITEEKYSLE
MSP3.4 C2    QRGNITTSQGNSHRATVVQQADQTNRLDNVNSVTQRGNNNYNNNLERGLGSGALPGTNIITEEKYSLE
MSP3.4 C3    QRGNITTSQGNSHRATVVQQADQTNRLDNVNSVTQRGNNNYNNNLERGLGSGALPGTNIITEEKYSLE
MSP3.4 C4    QRGNITTSQGNSHRATVVQQADQTNRLDNVNSVTQRGNNNYNNNLERGLGSGALPGTNIITEEKYSLE
MSP3.4 C5    QRGNITTSQGNSHRATVVQQADQTNRLDNVNSVTQRGNNNYNNNLERGLGSGALPGTNIITEEKYSLE
MSP3.4 C6    QRGNITTSQGNSHRATVVQQADQTNRLDNVNSVTQRGNNNYNNNLERGLGSGALPGTNIITEEKYSLE
MSP3.4 C7    QRGNITTSQGNSHRATVVQQADQTNRLDNVNSVTQRGNNNYNNNLERGLGSGALPGTNIITEEKYSLE
MSP3.4 C9    QRGNITTSQGNSHRATVVQQADQTNRLDNVNSVTQRGNNNYNNNLERGLGSGALPGTNIITEEKYSLE
MSP3.4 C10   QRGNITTSQGNSHRATVVQQADQTNRLDNVNSVTQRGNNNYNNNLERGLGSGALPGTNIITEEKYSLE
MSP3.4 C11   QRGNITTSQGNSHRATVVQQADQTNRLDNVNSVTQRGNNNYNNNLERGLGSGALPGTNIITEEKYSLE
MSP3.4 C12   QRGNITTSQGNSHRATVVQQADQTNRLDNVNSVTQRGNNNYNNNLERGLGSGALPGTNIITEEKYSLE
MSP3.4 D1    QRGNITTSQGNSHRATVVQQADQTNRLDNVNSVTQRGNNNYNNNLERGLGSGALPGTNIITEEKYSLE
MSP3.4 D3    QRGNITTSQGNSHRATVVQQADQTNRLDNVNSVTQRGNNNYNNNLERGLGSGALPGTNIITEEKYSLE
MSP3.4 D4    QRGNITTSQGNSHRATVVQQADQTNRLDNVNSVTQRGNNNYNNNLERGLGSGALPGTNIITEEKYSLE
MSP3.4 D5    QRGNITTSQGNSHRATVVQQADQTNRLDNVNSVTQRGNNNYNNNLERGLGSGALPGTNIITEEKYSLE
MSP3.4 D6    QRGNITTSQGNSHRATVVQQADQTNRLDNVNSVTQRGNNNYNNNLERGLGSGALPGTNIITEEKYSLE
MSP3.4 D7    QRGNITTSQGNSHRATVVQQADQTNRLDNVNSVTQRGNNNYNNNLERGLGSGALPGTNIITEEKYSLE
MSP3.4 D8    QRGNITTSQGNSHRATVVQQADQTNRLDNVNSVTQRGNNNYNNNLERGLGSGALPGTNIITEEKYSLE
MSP3.4 D9    QRGNITTSQGNSHRATVVQQADQTNRLDNVNSVTQRGNNNYNNNLERGLGSGALPGTNIITEEKYSLE
MSP3.4 D10   QRGNITTSQGNSHRATVVQQADQTNRLDNVNSVTQRGNNNYNNNLERGLGSGALPGTNIITEEKYSLE
MSP3.4 D11   QRGNITTSQGNSHRATVVQQADQTNRLDNVNSVTQRGNNNYNNNLERGLGSGALPGTNIITEEKYSLE
MSP3.4 D12   QRGNITTSQGNSHRATVVQQADQTNRLDNVNSVTQRGNNNYNNNLERGLGSGALPGTNIITEEKYSLE
MSP3.4 T1    QRGNITTSQGNSHRATVVQQADQTNRLDNVNSVTQRGNNNYNNNLERGLGSGALPGTNIITEEKYSLE
MSP3.4 T2    QRGNITTSQGNSHRATVVQQADQTNRLDNVNSVTQRGNNNYNNNLERGLGSGALPGTNIITEEKYSLE
MSP3.4 T3    QRGNITTSQGNSHRATVVQQADQTNRLDNVNSVTQRGNNNYNNNLERGLGSGALPGTNIITEEKYSLE
MSP3.4 T4    QRGNITTSQGNSHRATVVQQADQTNRLDNVNSVTQRGNNNYNNNLERGLGSGALPGTNIITEEKYSLE
MSP3.4 T5    QRGNITTSQGNSHRATVVQQADQTNRLDNVNSVTQRGNNNYNNNLERGLGSGALPGTNIITEEKYSLE
```

```
              70        80        90        100       110       120       130
               |         |         |         |         |         |         |
Contig# 1    ...........................................................      .|
MSP3.4 3D7   LIKLTSKDEEDIIKHNEDVREEIEEQQEDIEEDEEELENEGEETKEEDDEEKNETNDTEDTDDTEDTE
MSP3.4 B2    LIKLTSKDEEDIIKHNEDVREEIEEQQEDIEEDEEELENEGEETKEEDDEEKNETNDTEDTDDTEDTE
MSP3.4 B3    LIKLTSKDEEDIIKHNEDVREEIEEQQEDIEEDEEELENEGEETKEEDDEEKNETNDTEDTDDTEDTE
MSP3.4 B4    LIKLTSKDEEDIIKHNEDVREEIEEQQEDIEEDEEELENEGEETKEEDDEEKNETNDTEDTDDTEDTE
MSP3.4 B5    LIKLTSKDEEDIIKHNEDVREEIEEQQEDIEEDEEELENEGEETKEEDDEEKNETNDTEDTDDTEDTE
MSP3.4 B6    LIKLTSKDEEDIIKHNEDVREEIEEQQEDIEEDEEELENEGEETKEEDDEEKNETNDTEDTDDTEDTE
MSP3.4 B8    LIKLTSKDEEDIIKHNEDVREEIEEQQEDIEEDEEELENEGEETKEEDDEEKNETNDTEDTDDTEDTE
MSP3.4 B9    LIKLTSKDEEDIIKHNEDVREEIEEQQEDIEEDEEELENEGEETKEEDDEEKNETNDTEDTDDTEDTE
MSP3.4 B10   LIKLTSKDEEDIIKHNEDVREEIEEQQEDIEEDEEELENEGEETKEEDDEEKNETNDTEDTDDTEDTE
MSP3.4 B11   LIKLTSKDEEDIIKHNEDVREEIEEQQEDIEEDEEELENEGEETKEEDDEEKNETNDTEDTDDTEDTE
MSP3.4 B12   LIKLTSKDEEDIIKHNEDVREEIEEQQEDIEEDEEELENEGEETKEEDDEEKNETNDTEDTDDTEDTE
MSP3.4 C1    LIKLTSKDEEDIIKHNEDVREEIEEQQEDIEEDEEELENEGEETKEEDDEEKNETNDTEDTDDTEDTE
MSP3.4 C2    LIKLTSKDEEDIIKHNEDVREEIEEQQEDIEEDEEELENEGEETKEEDDEEKNETNDTEDTDDTEDTE
MSP3.4 C3    LIKLTSKDEEDIIKHNEDVREEIEEQQEDIEEDEEELENEGEETKEEDDEEKNETNDTEDTDDTEDTE
MSP3.4 C4    LIKLTSKDEEDIIKHNEDVREEIEEQQEDIEEDEEELENEGEETKEEDDEEKNETNDTEDTDDTEDTE
MSP3.4 C5    LIKLTSKDEEDIIKHNEDVREEIEEQQEDIEEDEEELENEGEETKEEDDEEKNETNDTEDTDDTEDTE
MSP3.4 C6    LIKLTSKDEEDIIKHNEDVREEIEEQQEDIEEDEEELENEGEETKEEDDEEKNETNDTEDTDDTEDTE
MSP3.4 C7    LIKLTSKDEEDIIKHNEDVREEIEEQQEDIEEDEEELENEGEETKEEDDEEKNETNDTEDTDDTEDTE
MSP3.4 C9    LIKLTSKDEEDIIKHNEDVREEIEEQQEDIEEDEEELENEGEETKEEDDEEKNETNDTEDTDDTEDTE
MSP3.4 C10   LIKLTSKDEEDIIKHNEDVREEIEEQQEDIEEDEEELENEGEETKEEDDEEKNETNDTED------TD
MSP3.4 C11   LIKLTSKDEEDIIKHNEDVREEIEEQQEDIEEDEEELENEGEETKEEDDEEKNETNDTEDTDDTEDTE
MSP3.4 C12   LIKLTSKDEEDIIKHNEDVREEIEEQQEDIEEDEEELENEGEETKEEDDEEKNETNDTEDTDDTEDTE
MSP3.4 D1    LIKLTSKDEEDIIKHNEDVREEIEEQQEDIEEDEEELENEGEETKEEDDEEKNETNDTEDTDDTEDTE
MSP3.4 D3    LIKLTSKDEEDIIKHNEDVREEIEEQQEDIEEDEEELENEGEETKEEDDEEKNETNDTEDTDDTEDTE
MSP3.4 D4    LIKLTSKDEEDIIKHNEDVREEIEEQQEDIEEDEEELENEGEETKEEDDEEKNETNDTEDTDDTEDTE
MSP3.4 D5    LIKLTSKDEEDIIKHNEDVREEIEEQQEDIEEDEEELENEGEETKEEDDEEKNETNDTEDTDDTEDTE
MSP3.4 D6    LIKLTSKDEEDIIKHNEDVREEIEEQQEDIEEDEEELENEGEETKEEDDEEKNETNDTEDTDDTEDTE
MSP3.4 D7    LIKLTSKDEEDIIKHNEDVREEIEEQQEDIEEDEEELENEGEETKEEDDEEKNETNDTEDTDDTEDTE
MSP3.4 D8    LIKLTSKDEEDIIKHNEDVREEIEEQQEDIEEDEEELENEGEETKEEDDEEKNETNDTED------TE
MSP3.4 D9    LIKLTSKDEEDIIKHNEDVREEIEEQQEDIEEDEEELENEGEETKEEDDEEKNETNDTEDTDDTEDTE
MSP3.4 D10   LIKLTSKDEEDIIKHNEDVREEIEEQQEDIEEDEEELENEGEETKEEDDEEKNETNDTED------TE
MSP3.4 D11   LIKLTSKDEEDIIKHNEDVREEIEEQQEDIEEDEEELENEGEETKEEDDEEKNETNDTEDTDDTEDTE
MSP3.4 D12   LIKLTSKDEEDIIKHNEDVREEIEEQQEDIEEDEEELENEGEETKEEDDEEKNETNDTEDTDDTEDTE
MSP3.4 T1    LIKLTSKDEEDIIKHNEDVREEIEEQQEDIEEDEEELENEGEETKEEDDEEKNETNDTEDTDDTEDTE
MSP3.4 T2    LIKLTSKDEEDIIKHNEDVREEIEEQQEDIEEDEEELENEGEETKEEDDEEKNETNDTED------TE
MSP3.4 T3    LIKLTSKDEEDIIKHNEDVREEIEEQQEDIEEDEEELENEGEETKEEDDEEKNETNDTEDTDDTEDTE
MSP3.4 T4    LIKLTSKDEEDIIKHNEDVREEIEEQQEDIEEDEEELENEGEETKEEDDEEKNETNDTEDTDDTEDTE
MSP3.4 T5    LIKLTSKDEEDIIKHNEDVREEIEEQQEDIEEDEEELENEGEETKEEDDEEKNETNDTEDTDDTEDTE
```

```
       140       150       160       170       180       190
                  |         |         |         |         |         |
Contig# 1    •••••• ••••••••••••••••••••••••••••••••••• ••••••••••••••••••••
MSP3.4 3D7   DIEEENKEKELSNQQQSEKKSISKVDEDSYRILSVSYKDNNEVKNVAESIVKKLFSLFNDNNN
MSP3.4 B2    DIEEENEEKELSNQQQSEKKSISKVDEDSYRILSVSYKDNNEVKNVAESIVKKLFSLFNDNNN
MSP3.4 B3    DIEEENEEKELSNQQQSEKKSISKVDEDSYRILSVSYKDNNEVKNVAESIVKKLF
MSP3.4 B4    DIEEENKEKELSNQQQSEKKSISKVDEDSYRIL
MSP3.4 B5    DIEEENKEKELSNQQQSEKKSISKVDEDSYRIL
MSP3.4 B6    DIEEENKEKELSNQQQSEKKSISKVDEDSYRILSVSYKDNNEVKNVAESIVKKL
MSP3.4 B8    DIEEENKEKELSNQQQSEKKSISKVDEDSYRILSVSYKDNNEVKNVAESIVKKLFSLFNDNNN
MSP3.4 B9    DIEEENEEKELSNQQQSEKKSISKVDEDSYRILSVSYKDNNEVKNVAESIVKKLFSLFNDNNN
MSP3.4 B10   DIEEENKEKELSNQQQSEKKSISKVDEDSYRILSVSYKDNNEVKNVAESIVKKLFSLFNDNNN
MSP3.4 B11   DIEEENEEKELSNQQQSEKKSISKVDEDSYRILSVSYKDNNEVKNVAESIVKKLFSLFNDNNN
MSP3.4 B12   DIEEENEEKELSNQQQSEKKSISKVDEDSYRILSVSYKDNNEVKNVAESIVKKLFSLFNDNNN
MSP3.4 C1    DIEEENKEKELSNQQQSEKKSISKVDEDSYRILSVSYKDNNEVKNVAESIVKKLFSLFNDNNN
MSP3.4 C2    DIEEENKEKELSNQQQSEKKSISKVDEDSYRILSVSYKDNNEVKNVAESIVKKLFSLFNDNNN
MSP3.4 C3    DIEEENEEKELSNQQQSEKKSISKVDEDSYRILSVSYKDNNEVKNVAESIVKKLFSLFNDNNN
MSP3.4 C4    DIEEENKEKELSNQQQSEKKSISKVDEDSYRILSVSYKDNNEVKNVAESIVKKLFSLFNDNNN
MSP3.4 C5    DIEEENKEKELSNQQQSEKKSISKVDEDSYRILSVSYKDNNEVKNVAESIVKKLFSLFNDNNN
MSP3.4 C6    DIEEENEEKELSNQQQSEKKSISKVDEDSYRILSVSYKDNNEVKNVAESIVKKLFSLFNDNNN
MSP3.4 C7    DIEEENKEKELSNQQQSEKKSISKVDEDSYRILSVSYKDNNEVKNVAESIVKKLFSLFNDNNN
MSP3.4 C9    DIEEENKEKELSNQQQSEKKSISKVDEDSYRILSVSYKDNNEVKNVAESIVKKLFSLFNDNNN
MSP3.4 C10   DIEEENKEKELSNQQQSEKKSISKVDEDSYRILSVSYKDNNEVKNVAESIVKKLFSLFNDNNN
MSP3.4 C11   DIEEENKEKELSNQQQSEKKSISKVDEDSYRILSVSYKDNNEVKNVAESIVKKLFSLFNDNNN
MSP3.4 C12   DIEEENKEKELSNQQQSEKKSISKVDEDSYRILSVSYKDNNEVKNVAESIVKKLFSLFNDNNN
MSP3.4 D1    DIEEENKEKELSNQQQSEKKSISKVDEDSYRILSVSYKDNNEVKNVAESIVKKLFSLFNDNNNL
MSP3.4 D3    DIEEENKEKELSNQQQSEKKSISKVDEDSYRILSVSYKDNNEVKNVAESIVKKLFSLFNDNNN
MSP3.4 D4    DIEEENKEKELSNQQQSEKKSISKVDEDSYRILSVSYKDNNEVKNVAESIVKKLFSLFNDNNN
MSP3.4 D5    DIEEENKEKELSNQQQSEKKSISKVDEDSYRILSVSYKDNNEVKNVAESIVKKLFSLFNDNNN
MSP3.4 D6    DIEEENEEKELSNQQQSEKKSISKVDEDSYRILSVSYKDNNEVKNVAESIVKKLFSLFNDNNN
MSP3.4 D7    DIEEENKEKELSNQQQSEKKSISKVDEDSYRILSVSYKDNNEVKNVAESIVKKLFSLFNDNNN
MSP3.4 D8    DIEEENEEKELSNQQQSEKKSISKVDEDSYRILSVSYKDNNEVKNVAESIVKKLFSLFNDNNN
MSP3.4 D9    DIEEENKEKELSNQQQSEKKSISKVDEDSYRILSVSYKDNNEAKNVAESIVKKLFSLFNDNNN
MSP3.4 D10   DIEEENEEKELSNQQQSEKKSISKVDEDSYRILSVSYKDNNEVKNVAESIVKKLFSLFNDNNN
MSP3.4 D11   DIEEENKEKELSNQQQSEKKSISKVDEDSYRILSVSYKDNNEVKNVAESIVKKLFSLFNDNNN
MSP3.4 D12   DIEEENKEKELSNQQQSEKKSISKVDEDSYRILSVSYKDNNEVKNVAESIVKKLFSLFNDNNN
MSP3.4 T1    DIEEENEEKELSNQQQSEKKSISKVDEDSYRILSVSYKDNNEVKNVAESIVKKLFSLFNDNNN
MSP3.4 T2    DIEEENEEKELSNQQQSEKKSISKVDEDSYRILSVSYKDNNEVKNVAESIVKKLFSLFNDNNN
MSP3.4 T3    DIEEENEEKELSNQQQSEKKSISKVDEDSYRILSVSYKDNNEVKNVAESIVKKLFSLFNDNNN
MSP3.4 T4    DIEEENEEKELSNQQQSEKKSISKVDEDSYRILSVSYKDNNEVKNVAESIVKKLFSLFNDNNN
MSP3.4 T5    DIEEENKEKELSNQQQSEKKSISKVDEDSYRILSVSYKDNNEVKNVAESIVKKLF
```

MSP3.7 C-term amino acid alignments:

```
                    10        20        30        40        50        60
                     |         |         |         |         |         |
Contig# 1             ........................|...............................
MSP3.7 3D7   PYNHYFAWEIGGGAPTYKPENNKNDNILLEHVKITSWDKEDIIKENEDTKREVQETEDTDETEDTDET
MSP3.7 B1    PYNHYFAWEIGGGAPTYKPENNKNDNILLEHVKITSWDKEDIIKENEDTKREVQETEDTDETEDTDET
MSP3.7 B2    PYNHYFAWEIGGGAPTYKPENNKNDNILLEHVKITSWDKEDIIKENEDTKREVQETEDTDETEDTDET
MSP3.7 B3    PYNHYFAWEIGGGAPTYKPENNKNDNILLEHVKITPWDKEDIIKENEDTKREVQETEDTDETEDTDET
MSP3.7 B4    PYNHYFAWEIGGGAPTYKPENNKNDNILLEHVKITSWDKEDIIKENEDTKREVQETEDTDETEDTDET
MSP3.7 B5    PYNHYFAWEIGGGAPTYKPENNKNDNILLEHVKITSWDKEDIIKENEDTKREVQETEDTDETEDTDET
MSP3.7 B8    PYNHYFAWEIGGGAPTYKPENNKNDNILLEHVKITSWDKEDIIKENEDTKREVQETEDTDETEDTDET
MSP3.7 B9    ----YFAWEIGGGAPTYKPENNKNDNILLEHVKITSWDKEDIIKENEDTKREVQETEDTDETEDTDET
MSP3.7 B10   -YNHYFAWEIGGGAPTYKPENNKNDNILLEHVKITSWDKEDIIKENEDTKREVQETEDTDETEDTDET
MSP3.7 B11   -----------GGAPTYKPENNKNDNILLEHVKITSWDKEDIIKENEDTKREVQETEDTDETEDTDET
MSP3.7 B12   PYNHYFAWEIGGGAPTYKPENNKNDNILLEHVKITSWDKEDIIKENEDTKREVQETEDTDETEDTDET
MSP3.7 C1    PYNHYFAWEIGGGAPTYKPENNKNDNILLEHVKITSWDKEDIIKENEDTKREVQETEDTDETEDTDET
MSP3.7 C2    PYNHYFAWEIGGGAPTYKPENNKNDNILLEHVKITSWDKEDIIKENEDTKREVQETEDTDETEDTDET
MSP3.7 C3    PYNHYFAWEIGGGAPTYKPENNKNDNILLEHVKITSWDKEDIIKENEDTKREVQETEDTDETEDTDET
MSP3.7 C4    PYNHYFAWEIGGGAPTYKPENNKNDNILLEHVKITSWDKEDIIKENEDTKREVQETEDTDETEDTDET
MSP3.7 C5    PYNHYFAWEIGGGAPTYKPENNKNDNILLEHVKITSWDKEDIIKENEDTKREVQETEDTDETEDTDET
MSP3.7 C6    -YNHYFAWEIGGGAPTYKPENNKNDNILLEHVKITSWDKEDIIKENEDTKREVQETEDTDETEDTDET
MSP3.7 C7    PYNHYFAWEIGGGAPTYKPENNKNDNILLEHVKITSWDKEDIIKENEDTKREVQETEDTDETEDTDET
MSP3.7 C9    -YNHYFAWEIGGGAPTYKPENNKNDNILLEHVKITSWDKEDIIKENEDTKREVQETEDTDETEDTDET
MSP3.7 C10   PYNHYFAWEIGGGAPTYKPENNKNDNILLEHVKITSWDKEDIIKENEDTKREVQETEDTDETEDTDET
MSP3.7 C11   PYNHYFAWEIGGGAPTYKPENNKNDNILLEHVKITSWDKEDIIKENEDTKREVQETEDTDETEDTDET
MSP3.7 C12   PYNHYFAWEIGGGAPTYKPENNKNDNILLEHVKITSWDKEDIIKENEDTKREVQETEDTDETEDTDET
MSP3.7 D1    PYNHYFAWEIGGGAPTYKPENNKNDNILLEHVKITSWDKEDIIKENEDTKREVQETEDTDETEDTDET
MSP3.7 D2    PYNHYFAWEIGGGAPTYKPENNKNDNILLEHVKITSWDKEDIIKENEDTKREVQETEDTDETEDTDET
MSP3.7 D5    PYNHYFAWEIGGGAPTYKPENNKNDNILLEHVKITSWDKEDIIKENEDTKREVQETEDTDETEDTDET
MSP3.7 D6    PYNHYFAWEIGGGAPTYKPENNKNDNILLEHVKITSWDKEDIIKENEDTKREVQETEDTDETEDTDET
MSP3.7 D7    PYNHYFAWEIGGGAPTYKPENNKNDNILLEHVKITSWDKEDIIKENEDTKREVQETEDTDETEDTDET
MSP3.7 D8    PYNHYFAWEIGGGAPTYKPENNKNDNILLEHVKITSWDKEDIIKENEDTKREVQETEDTDETEDTDET
MSP3.7 D9    PYNHYFAWEIGGGAPTYKPENNKNDNILLEHVKITSWDKEDIIKENEDTKREVQETEDTDETEDTDET
MSP3.7 D10   PYNHYFAWEIGGGAPTYKPENNKNDNILLEHVKITSWDKEDIIKENEDTKREVQETEDTDETEDTDET
MSP3.7 D11   PYNHYFAWEIGGGAPTYKPENNKNDNILLEHVKITSWDKEDIIKENEDTKREVQETEDTDETEDTDET
MSP3.7 D12   -YNHYFAWEIGGGAPTYKPENNKNDNILLEHVKITSWDKEDIIKENEDTKREVQETEDTDETEDTDET
MSP3.7 D13   PYNHYFAWEIGGGAPTYKPENNKNDNILLEHVKITSWDKEDIIKENEDTKREVQETEDTDETEDTDET
MSP3.7 T1    PYNHYFAWEIGGGAPTYKPENNKNDNILLEHVKITSWDKEDIIKENEDTKREVQETEDTDETEDTDET
MSP3.7 T2    PYNHYFAWEIGGGAPTYKPENNKNDNILLEHVKITSWDKEDIIKENEDTKREVQETEDTDETEDTDET
MSP3.7 T3    PYNHYFAWEIGGGAPTYKPENNKNDNILLEHVKITSWDKEDIIKENEDTKREVQETEDTDETEDTDET
MSP3.7 T4    PYNHYFAWEIGGGAPTYKPENNKNDNILLEHVKITSWDKEDIIKENEDTKREVQETEDTDETEDTDET
MSP3.7 T5    PYNHYFAWEIGGGAPTYKPENNKNDNILLEHVKITSWDKEDIIKENEDTKREVQETEDTDETEDTDET
```

```
               70        80        90        100       110       120       130
                |         |         |         |         |         |         |
Contig# 1      ....................................................................
MSP3.7 3D7     EETEDMEDENEIVEDQLQENEDDEDNVNLEDINKNTRNDIFEEQIKLDSTQDDKAQKLISNEYKKTEE
MSP3.7 B1      EETEDMEDENEIVEDQLQENEDDEDNVNLEDINKNTRNDIFEEQIKLDSTQDDKAQKLISNEYKKTEE
MSP3.7 B2      EETEDMEDENEIVEDQLQENEDDEDNVNLEDINKNTRNDIFEEQIKLDSTQDDKAQKLISNEYKKTEE
MSP3.7 B3      EETEDMEDENEIVEDQLQENEDDEDNVNLEDINKNTRNDIFEEQIKLDSTQDDKAQKLISNEYKKTEE
MSP3.7 B4      EETEDMEDENEIVEDQLQENEDDEDNVNLEDINKNTRNDIFEEQIKLDSTQDDKAQKLISNEYKKTEE
MSP3.7 B5      EETEDMEDENEIVEDQLQENEDDEDNVNLEDINKNTRNDIFEEQIKLDSTQDDKAQKLISNEYKKTEE
MSP3.7 B8      EETEDMEDENEIVEDQLQENEDDEDNVNLEDINKNTRNDIFEEQIKLDSTQDDKAQKLISNEYKKTEE
MSP3.7 B9      EETEDMEDENEIVEDQLQENEDDEDNVNLEDINKNTRNDIFEEQIKLDSTQDDKAQKLISNEYKKTEE
MSP3.7 B10     EETEDMEDENEIVEDQLQENEDDEDNVNLEDINKNTRNDIFEEQIKLDSTQDDKAQKLISNEYKKTEE
MSP3.7 B11     EETEDMEDENEIVEDQLQENEDDEDNVNLEDINKNTRNDIFEEQIKLDSTQDDKAQKLISNEYKKTEE
MSP3.7 B12     EETEDMEDENEIVEDQLQENEDDEDNVNLEDINKNTRNDIFEEQIKLDSTQDDKAQKLISNEYKKTEE
MSP3.7 C1      EETEDMEDENEIVEDQLQENEDDEDNVNLEDINKNTRNDIFEEQIKLDSTQDDKAQKLISNEYKKTEE
MSP3.7 C2      EETEDMEDENEIVEDQLQENEDDEDNVNLEDINKNTRNDIFEEQIKLDSTQDDKAQKLISNEYKKTEE
MSP3.7 C3      EETEDMEDENEIVEDQLQENEDDEDNVNLEDINKNTRNDIFEEQIKLDSTQDDKAQKLISNEYKKTEE
MSP3.7 C4      EETEDMEDENEIVEDQLQENEDDEDNVNLEDINKNTRNDIFEEQIKLDSTQDDKAQKLISNEYKKTEE
MSP3.7 C5      EETEDMEDENEIVEDQLQENEDDEDNVNLEDINKNTRNDIFEEQIKLDSTQDDKAQKLISNEYKKTEE
MSP3.7 C6      EETEDMEDENEIVEDQLQENEDDEDNVNLEDINKNTRNDIFEEQIKLDSTQDDKAQKLISNEYKKTEE
MSP3.7 C7      EETEDMEDENEIVEDQLQENEDDEDNVNLEDINKNTRNDIFEEQIKLDSTQDDKAQKLISNEYKKTEE
MSP3.7 C9      EETEDMEDENEIVEDQLQENEDDEDNVNLEDINKNTRNDIFEEQIKLDSTQDDKAQKLISNEYKKTEE
MSP3.7 C10     EETEDMEDENEIVEDQLQENEDDEDNVNLEDINKNTRNDIFEEQIKLDSTQDDKAQKLISNEYKKTEE
MSP3.7 C11     EETEDMEDENEIVEDQLQENEDDEDNVNLEDINKNTRNDIFEEQIKLDSTQDDKAQKLISNEYKKTEE
MSP3.7 C12     EETEDMEDENEIVEDQLQENEDDEDNVNLEDINKNTRNDIFEEQIKLDSTQDDKAQKLISNEYKKTEE
MSP3.7 D1      EETEDMEDENEIVEDQLQENEDDEDNVNLEDINKNTRNDIFEEQIKLDSTQDDKAQKLISNEYKKTEE
MSP3.7 D2      EETEDMEDENEIVEDQLQENEDDEDNVNLEDINKNTRNDIFEEQIKLDSTQDDKAQKLISNEYKKTEE
MSP3.7 D5      EETEDMEDENEIVEDQLQENEDDEDNVNLEDINKNTRNDIFEEQIKLDSTQDDKAQKLISNEYKKTEE
MSP3.7 D6      EETEDMEDENEIVEDQLQENEDDEDNVNLEDINKNTRNDIFEEQIKLDSTQDDKAQKLISNEYKKTEE
MSP3.7 D7      EETEDMEDENEIVEDQLQENEDDEDNVNLEDINKNTRNDIFEEQIKLDSTQDDKAQKLISNEYKKTEE
MSP3.7 D8      EETEDMEDENEIVEDQLQENEDDEDNVNLEDINKNTRNDIFEEQIKLDSTQDDKAQKLISNEYKKTEE
MSP3.7 D9      EETEDMEDENEIVEDQLQENEDDEDNVNLEDINKNTRNDIFEEQIKLDSTQDDKAQKLISNEYKKTEE
MSP3.7 D10     EETEDMEDENEIVEDQLQENEDDEDNVNLEDINKNTRNDIFEEQIKLDSTQDDKAQKLISNEYKKTEE
MSP3.7 D11     EETEDMEDENEIVEDQLQENEDDEDNVNLEDINKNTRNDIFEEQIKLDSTQDDKAQKLISNEYKKTEE
MSP3.7 D12     EETEDMEDENEIVEDQLQENEDDEDNVNLEDINKNTRNDIFEEQIKLDSTQDDKAQKLISNEYKKTEE
MSP3.7 D13     EETEDMEDENEIVEDQLQENEDDEDNVNLEDINKNTRNDIFEEQIKLDSTQDDKAQKLISNEYKKTEE
MSP3.7 T1      EETEDMEDENEIVEDQLQENEDDEDNVNLEDINKNTRNDIFEEQIKLDSTQDDKAQKLISNEYKKTEE
MSP3.7 T2      EETEDMEDENEIVEDQLQENEDDEDNVNLEDINKNTRNDIFEEQIKLDSTQDDKAQKLISNEYKKTEE
MSP3.7 T3      EETEDMEDENEIVEDQLQENEDDEDNVNLEDINKNTRNDIFEEQIKLDSTQDDKAQKLISNEYKKTEE
MSP3.7 T4      EETEDMEDENEIVEDQLQENEDDEDNVNLEDINKNTRNDIFEEQIKLDSTQDDKAQKLISNEYKKTEE
MSP3.7 T5      EETEDMEDENEIVEDQLQENEDDEDNVNLEDINKNTRNDIFEEQIKLDSTQDDKAQKLISNEYKKTEE
```

```
                140       150       160       170
                  |         |         |         |
Contig# 1    .......................................
MSP3.7 3D7   KKSLEDHVNLLFNFLQTNNQLDPSLKDLENELTFFLNNY
MSP3.7 B1    KKSLEDHVNLLFNFLQTNNQLDPSLKDLENELTFFLNNY
MSP3.7 B2    KKSLEDHVNLLFNFLQTNNQLDPSLKDLENELTFFLNNY
MSP3.7 B3    KKSLEDHVNLLFNFLQTNNQLDPSLKDLENELTFFLNNY
MSP3.7 B4    KKSLEDHVNLLFNFLQTNNQLDPSLKDLENELTFFLNNY
MSP3.7 B5    KKSLEDHVNLLFNFLQTNNQLDPSLKDLENELTFFLNNY
MSP3.7 B8    KKSLEDHVNLLFNFLQTNNQLDPSLKDLENELTFFLNNY
MSP3.7 B9    KKSLEDHVNLLFNFLQTNNQLDPSLKDLENELTFFLNNY
MSP3.7 B10   KKSLEDHVNLLFNFLQTNNQLDPSLKDLENELTFFLNNY
MSP3.7 B11   KKSLEDHVNLLFNFLQTNNQLDPSLKDLENELTFFLNNY
MSP3.7 B12   KKSLEDHVNLLFNFLQTNNQLDPSLKDLENELTFFLNNY
MSP3.7 C1    KKSLEDHVNLLFNFLQTNNQLDPSLKDLENELTFFLNNY
MSP3.7 C2    KKSLEDHVNLLFNFLQTNNQLDPSLKDLENELTFFLNNY
MSP3.7 C3    KKSLEDHVNLLFNFLQTNNQLDPSLKDLENELTFFLNNY
MSP3.7 C4    KKSLEDHVNLLFNFLQTNNQLDPSLKDLENELTFFLNNY
MSP3.7 C5    KKSLEDHVNLLFNFLQTNNQLDPSLKDLENELTFFLNNY
MSP3.7 C6    KKSLEDHVNLLFNFLQTNNQLDPSLKDLENELTFFLNNY
MSP3.7 C7    KKSLEDHVNLLFNFLQTNNQLDPSLKDLENELTFFLNNY
MSP3.7 C9    KKSLEDHVNLLFNFLQTNNQLDPSLKDLENELTFFLNNY
MSP3.7 C10   KKSLEDHVNLLFNFLQTNNQLDPSLKDLENELTFFLNNY
MSP3.7 C11   KKSLEDHVNLLFNFLQTNNQLDPSLKDLENELTFFLNNY
MSP3.7 C12   KKSLEDHVNLLFNFLQTNNQLDPSLKDLENELTFFLNNY
MSP3.7 D1    KKSLEDHVNLLFNFLQTNNQLDPSLKDLENELTFFLNNY
MSP3.7 D2    KKSLEDHVNLLFNFLQTNNQLDPSLKDLENELTFFLNNY
MSP3.7 D5    KKSLEDHVNLLFNFLQTNNQLDPSLKDLENELTFFLNNY
MSP3.7 D6    KKSLEDHVNLLFNFLQTNNQLDPSLKDLENELTFFLNNY
MSP3.7 D7    KKSLEDHVNLLFNFLQTNNQLDPSLKDLENELTFFLNNY
MSP3.7 D8    KKSLEDHVNLLFNFLQTNNQLDPSLKDLENELTFFLNNY
MSP3.7 D9    KKSLEDHVNLLFNFLQTNNQLDPSLKDLENELTFFLNNY
MSP3.7 D10   KKSLEDHVNLLFNFLQTNNQLDPSLKDLENELTFFLNNY
MSP3.7 D11   KKSLEDHVNLLFNFLQTNNQLDPSLKDLENELTFFLNNY
MSP3.7 D12   KKSLEDHVNLLFNFLQTNNQLDPSLKDLENELTFFLNNY
MSP3.7 D13   KKSLEDHVNLLFNFLQTNNQLDPSLKDLENELTFFLNNY
MSP3.7 T1    KKSLEDHVNLLFNFLQTNNQLDPSLKD
MSP3.7 T2    KKSLEDHVNLLFNFLQTNNQLDPSLKD
MSP3.7 T3    KKSLEDHVNLLFNFLQTNNQLDPS
MSP3.7 T4    KKSLEDHVNLLFNFLQTNNQLDPS
MSP3.7 T5    KKSLEDHVNLLFNFLQTNNQLDPS
```

MSP3.8 C-term amino acid alignments:

```
                       10        20        30        40        50        60
                        |         |         |         |         |         |
Contig# 1     ................................. |.............................
MSP3.8 3D7S   HSKTIDPSKIDDRLELSSGSSSLEQHSKEDVKKGCALELVPLSLSDIEQIANESEDVLEEIEEEIN
MSP3.8 B2     HSKTIDPSKIDDRLELSSGSSSLEQHSKEDVKKGSALELVPLSLSDIEQIANESEDVLEEIEEEIN
MSP3.8 B3     HSKTIDPSKIDDRLELSSGSSSLEQHSKEDVKKGSALELVPLSLSDIEQIANESEDVLEEIEEEIN
MSP3.8 B4     HSKTIDPSKIDDRLELSSGSSSLEQHSKEDVKKGSALELVPLSLSDIEQIANESEDVLEEIEEEIN
MSP3.8 B5     HSKTIDPSKIDDRLELSSGSSSLEQHSKEDVKKGSALELVPLSLSDIEQIANESEDVLEEIEEEIN
MSP3.8 B6     -SKTIDPSKIDDRLELSSGSSSLEQHSKEDVKKGSALELVPLSLSDIEQIANESEDVLEEIEEEIN
MSP3.8 B8     HSKTIDPSKIDDRLELSSGSSSLEQHSKEDVKKGSALELVPLSLSDIEQIANESEDVLEEIEEEIN
MSP3.8 B9     HSKTIDPSKIDDRLELSSGSSSLEQHSKEDVKKGSALELVPLSLSDIEQIANESEDVLEEIEEEIN
MSP3.8 B10    HSKTIDPSKIDDRLELSSGSSSLEQHSKEDVKKGSALELVPLSLSDIEQIANESEDVLEEIEEEIN
MSP3.8 B11    HSKTIDPSKIDDRLELSSGSSSLEQHSKEDVKKGSALELVPLSLSDIEQIANESEDVLEEIEEEIN
MSP3.8 B12    HSKTIDPSKIDDRLELSSGSSSLEQHSKEDVKKGSALELVPLSLSDIEQIANESEDVLEEIEEEIN
MSP3.8 C1     HSKTIDPSKIDDRLELSSGSSSLEQHSKEDVKKGSALELVPLSLSDIEQIANESEDVLEEIEEEIN
MSP3.8 C2     HSKTIDPSKIDDRLELSSGSSSLEQHSKEDVKKGSALELVPLSLSDIEQIANESEDVLEEIEEEIN
MSP3.8 C3     HSKTIDPSKIDDRLELSSGSSSLEQHSKEDVKKGSALELVPLSLSDIEQIANESEDVLEEIEEEIN
MSP3.8 C4     HSKTIDPSKIDDRLELSSGSSSLEQHSKEDVKKGSALELVPLSLSDIEQIANESEDVLEEIEEEIN
MSP3.8 C5     HSKTIDPSKIDDRLELSSGSSSLEQHSKEDVKKGSALELVPLSLSDIEQIANESEDVLEEIEEEIN
MSP3.8 C6     HSKTIDPSKIDDRLELSSGSSSLEQHSKEDVKKGSALELVPLSLSDIEQIANESEDVLEEIEEEIN
MSP3.8 C7     HSKTIDPSKIDDRLELSSGSSSLEQHSKEDVKKGSTLELVPLSLSDIEQIANESEDVLEEIEEEIN
MSP3.8 C9     HSKTIDPSKIDDRLELSSGSSSLEQHSKEDVKKGSALELVPLSLSDIEQIANESEDVLEEIEEEIN
MSP3.8 C10    HSKTIDPSKIDDRLELSSGSSSLEQHSKEDVKKGSALELVPLSLSDIEQIANESEDVLEEIEEEIN
MSP3.8 C12    HSKTIDPSKIDDRLELSSGSSSLEQHSKEDVKKGSALELVPLSLSDIEQIANESEDVLEEIEEEIN
MSP3.8 D1     HSKTIDPSKIDDRLELSSGSSSLEQHSKEDVKKGSALELVPLSLSDIEQIANESEDVLEEIEEEIN
MSP3.8 D2     HSKTIDPSKIDDRLELSSGSSSLEQHSKEDVKKGCALELVPLSLSDIEQIANESEDVLEEIEEEIN
MSP3.8 D3     HSKTIDPSKIDDRLELSSGSSSLEQHSKEDVKKGSALELVPLSLSDIEQIANESEDVLEEIEEEIN
MSP3.8 D5     HSKTIDPSKIDDRLELSSGSSSLEQHSKEDVKKGSALELVPLSLSDIEQIANESEDVLEEIEEEIN
MSP3.8 D6     HSKTIDPSKIDDRLELSSGSSSLEQHSKEDVKKGSALELVPLSLSDIEQIANESEDVLEEIEEEIN
MSP3.8 D7     HSKTIDPSKIDDRLELSSGSSSLEQHSKEDVKKGSALELVPLSLSDIEQIANESEDVLEEIEEEIN
MSP3.8 D9     HSKTIDPSKIDDRLELSSGSSSLEQHSKEDVKKGSALELVPLSLSDIEQIANESEDVLEEIEEEIN
MSP3.8 D10    HSKTIDPSKIDDRLELSSGSSSLEQHSKEDVKKGSALELVPLSLSDIEQIANESEDVLEEIEEEIN
MSP3.8 D11    HSKTIDPSKIDDRLELSSGSSSLEQHSKEDVKKGSALELVPLSLSDIEQIANESEDVLEEIEEEIN
MSP3.8 D12    HSKTIDPSKIDDRLELSSGSSSLEQHSKEDVKKGSALELVPLSLSDIEQIANESEDVLEEIEEEIN
MSP3.8 D13    HSKTIDPSKIDDRLELSSGSSSLEQHSKEDVKKGCALELVPLSLSDIEQIANESEDVLEEIEEEIN
MSP3.8 T1     HSKTIDPSKIDDRLELSSGSSSLEQHSKEDVKKGSALELVPLSLSDIEQIANESEDVLEEIEEEIN
MSP3.8 T2     HSKTIDPSKIDDRLELSSGSSSLEQHSKEDVKKGSALELVPLSLSDIEQIANESEDVLEEIEEEIN
MSP3.8 T3     HSKTIDPSKIDDRLELSSGSSSLEQHSKEDVKKGSALELVPLSLSDIEQIANESEDVLEEIEEEIN
MSP3.8 T4     HSKTIDPSKIDDRLELSSGSSSLEQHSKEDVKKGSALELVPLSLSDIEQIANESEDVLEEIEEEIN
MSP3.8 T5     HSKTIDPSKIDDRLELSSGSSSLEQHSKEDVKKGSALELVPLSLSDIEQIANESEDVLEEIEEEIN
```

```
                 70        80        90        100       110       120       130
                 |         |         |         |         |         |         |
Contig# 1
MSP3.8 3D7S   TDGEIEYITEEEIKEDIEEETEEDIEEETEEETEEETEEE--------ADEETVKEIEDKPEQEIK
MSP3.8 B2     TDGEIEYITEEEIKEDIEEETGEDIEEETEEETEEETEEE--------ADEETVKEIEDKPEQEIK
MSP3.8 B3     TDGEIEYITEEEIKEDIEEETGEDIEEETEEETEEETEEE--------ADEETVKEIEDKPEQEIK
MSP3.8 B4     TDGEIEYITEEEIKEDIEEETEEDIEEETEEETEEETEEETEEE----ADEETVKEIEDKPEQEIK
MSP3.8 B5     TDGEIEYITEEEIKEDIEEETEEDIEEETEEETEEETEEETEEE----ADEETVKEIEDKPEQEIK
MSP3.8 B6     TDGEIEYITEEEIKEDIEEEIKEDIEEETEEDIEEETEEETEEE----ADEETVKEIEDKPEQEIK
MSP3.8 B8     TDGEIEYITEEEIKEDIEEEIKEDIEEETEEDIEEETEEETEEE----ADEETVKEIEDKPEQEIK
MSP3.8 B9     TDGEIEYITEEEIKEDIEEETGEDIEEETEEETEEETEEE--------ADEETVKEIEDKPEQEIK
MSP3.8 B10    TDGEIEYITEEEIKEDIEEEIKEDIEEETEEDIEEETEEETEEE----ADEETVKEIEDKPEQEIK
MSP3.8 B11    TDGEIEYITEEEIKEDIEEETGEDIEEETEEETEEETEEE--------ADEETVKEIEDKPEQEIK
MSP3.8 B12    TDGEIEYITEEEIKEDIEEETGEDIEEETEEETEEETEEE--------ADEETVKEIEDKPEQEIK
MSP3.8 C1     TDGEIEYITEEEIKEDIEEETEEDIEEETEEETEEEAEEE--------ADEETVKEIEDKPEQEIK
MSP3.8 C2     TDGEIEYITEEEIKEDIEEETEEDIEEETEEETEEETEEE--------ADEETVKEIEDKPEQEIK
MSP3.8 C3     TDGEIEYITEEEIKEDIEEETEEDIEEETEEETEEETEEE--------ADEETVKEIEDKPEQEIK
MSP3.8 C4     TDGEIEYITEEEIKEDIEEETEEDIEEETEEETEEETEEETEEETEEEADEETVKEIEDKPEQEIK
MSP3.8 C5     TDGEIEYITEEEIKEDIEEETEEDIEEETEEETEEETEEETEEETEEEADEETVKEIEDKPEQEIK
MSP3.8 C6     TDGEIEYITEEEIKEDIEEETEEDIEEETEEETEEETEEE--------ADEETVKEIEDKPEQEIK
MSP3.8 C7     TDGEIEYITEEEIKEDIEEETEEDIEEETEEETEEETEEE--------ADEETVKEIEDKPEQEIK
MSP3.8 C9     TDGEIEYITEEEIKEDIEEETEEDIEEETEEETEEETEEE--------ADEETVKEIEDKPEQEIK
MSP3.8 C10    TDGEIEYITEEEIKEDIEEETEEDIEEETEEETEEETEEETEEE----ADEETVKEIEDKPEQEIK
MSP3.8 C12    TDGEIEYITEEEIKEDIEEETEEDIEEETEEETEEETEEE--------ADEETVKEIEDKPEQEIK
MSP3.8 D1     TDGEIEYITEEEIKEDIEEETEEDIEEETEEETEEETEEETEEE----ADEETVKEIEDKPEQEIK
MSP3.8 D2     TDGEIEYITEEEIKEDIEEETEEDIEEETEEETEEETEEE--------ADEETVKEIEDKPEQEIK
MSP3.8 D3     TDGEIEYITEEEIKEDIEEETEEDIEEETEEETEEETEEETEEE----ADEETVKEIEDKPEQEIK
MSP3.8 D5     TDGEIEYITEEEIKEDIEEETEEDIEEETEEETEEETEEE--------ADEETVKEIEDKPEQEIK
MSP3.8 D6     TDGEIEYITEEEIKEDIEEETEEDIEEETEEETEEETEEETEEE----ADEETVKEIEDKPEQEIK
MSP3.8 D7     TDGEIEYITEEEIKEDIEEETEEDIEEETEEETEEETEEETEEE----ADEETVKEIEDKPEQEIK
MSP3.8 D9     TDGEIEYITDEEIKEDIEEETEEDIEEETEEETEEETEEETEEE----ADEETVKEIEDKPEQEIK
MSP3.8 D10    TDGEIEYITDEEIKEDIEEETEEDIEEETEEETEEETEEETEEE----ADEETVKEIEDKPEQEIK
MSP3.8 D11    TDGEIEYITDEEIKEDIEEETEEDIEEETEEETEEETEEETEEE----ADEETVKEIEDKPEQEIK
MSP3.8 D12    TDGEIEYITDEEIKEDIEEETEEDIEEETEEETEEETEEET----EEEADEETVEEIEDEPEQEIK
MSP3.8 D13    TDGEIEYITEEEIKEDIEEETEEDIEEETEEETEEETEEE--------ADEETVKEIEDKPEQEIK
MSP3.8 T1     TDGEIEYITEEEIKEDIEEETEEDIEEETEEETEEETEEE--------ADEETVKEIEDKPEQEIK
MSP3.8 T2     TDGEIEYITEEEIKEDIEEETEEDIEEETEEETEEETEEET----EEEADEETVKVIEDKPEQEIK
MSP3.8 T3     TDGEIEYITDEEIKEDIEEETEEDIEEETEEETEEETEEE--------ADEETVKEIEDKPEQEIK
MSP3.8 T4     TDGEIEYITEEEIKEDIEEEIKEDIEEETEEDIEEETEEETEEETEEEADEETVKEIEDKPEQEIK
MSP3.8 T5     TDGEIEYITEEEIKEDIEEETEEDIEEETEEETEEETEEE--------ADEETVKEIEDKPEQEIK
```

```
                   140       150       160       170       180       190
                     |         |         |         |         |         |
Contig# 1     . ..... .................|.................................... ...
MSP3.8 3D7S   NKSLEEKQIDKNTDTSEKKGFNNSEKDEKARNLISKNYKNYNELDKNVHTLVNSIISLLEEGNGSD
MSP3.8 B2     NKSLEEKQIDKNTDTSEKKGFNNSEKDEKARNLISKNYKNYNELDKNVHTLVNSIISLLEEGNGSD
MSP3.8 B3     NKSLEEKQIDKNTDTSEKKGFNNSEKDEKARNLISKNYKNYNELDKNVHTLVNSIISLLEEGNGSD
MSP3.8 B4     NKSLEEKQIDKNTDTSEKKGFNNSEKDEKARNLISKNYKNYNELDKNVHTLVNSIISLLEEGNGSD
MSP3.8 B5     NKSLEEKQIDKNTDTSEKKGFNNSEKDEKARNLISKNYKNYNELDKNVHTLVNSIISLLEEGNGSD
MSP3.8 B6     NKSLEEKQIDKNTDTSEKKGFNNSEKDEKARNLISKNYKNYNELDKNVHTLVNSIISLLEEGNGSD
MSP3.8 B8     NKSLEEKQIDKNTDTSEKKGFNNSEKDEKARNLISKNYKNYNELDKNVHTLVNSIISLLEEGNGSD
MSP3.8 B9     NKSLEEKQIDKNTDTSEKKGFNNSEKDEKARNLISKNYKNYNELDKNVHTLVNSIISLLEEGNGSD
MSP3.8 B10    NKSLEEKQIDKNTDTSEKKGFNNSEKDEKARNLISKNYKNYNELDKNVHTLVNSIISLLEEGNGSD
MSP3.8 B11    NKSLEEKQIDKNTDTSEKKGFNNSEKDEKARNLISKNYKNYNELDKNVHTLVNSIISLLEEGNGSD
MSP3.8 B12    NKSLEEKQIDKNTDTSEKKGFNNSEKDEKARNLISKNYKNYNELDKNVHTLVNSIISLLEEGNGSD
MSP3.8 C1     NKSLEEKQIDKNTDTSEKKGFNNSEKDEKARNLISKNYKNYNELDKNVHTLVNSIISLLEEGNGSD
MSP3.8 C2     NKSLEEKQIDKNTDTSEKKGFNNSEKDEKARNLISKNYKNYNELDKNVHTLVNSIISLLEEGNGSD
MSP3.8 C3     NKSLEEKQIDKNTDTSEKKGFNNSEKDEKARNLISKNYKNYNELDKNVHTLVNSIISLLEEGNGSD
MSP3.8 C4     NKSLEEKQIDKNTDTSEKKGFNNSEKDEKARNLISKNYKNYNELDKNVHTLVNSIISLLEEGNGSD
MSP3.8 C5     NKSLEEKQIDKNTDTSEKKGFNNSEKDEKARNLISKNYKNYNELDKNVHTLVNSIISLLEEGNGSD
MSP3.8 C6     NKSLEEKQIDKNTDTSEKKGFNNSEKDEKARNLISKNYKNYNELDKNVHTLVNSIISLLEEGNGSD
MSP3.8 C7     NKSLEEKLIDKNTDTSEKKGFNNSEKDEKARNLISKNYKNYNELDKNVHTLVNSIISLLEEGNGSD
MSP3.8 C9     NKSLEEKQIDKNTDTSEKKGFNNSEKDEKARNLISKNYKNYNELDKNVHTLVNSIISLLEEGNGSD
MSP3.8 C10    NKSLEEKQIDKNTDTSEKKGFNNSEKDEKARNLISKNYKNYNELDKNVHTLVNSIISLLEEGNGSD
MSP3.8 C12    NKSLEEKQIDKNTDTSEKKGFNNSEKDEKARNLISKNYKNYNELDKNVHTLVNSIISLLEEGNGSD
MSP3.8 D1     NKSLEEKQIDKNTDTSEKKGFNNSEKDEKARNLISKNYKNYNELDKNVHTLVNSIISLLEEGNGSD
MSP3.8 D2     NKSLEEKLIDKNTDTSEKKGFNNSEKDEKARNLISKNYKNYNELDKNVHTLVNSIISLLEEGNGSD
MSP3.8 D3     NKSLEEKQIDKNTDTSEKKGFNNSEKDEKARNLISKNYKNYNELDKNVHTLVNSIISLLEEGNGSD
MSP3.8 D5     NKSLEEKQIDKNTDTSEKKGFNNSEKDEKARNLISKNYKNYNELDKNVHTLVNSIISLLEEGNGSD
MSP3.8 D6     NKSLEEKQIDKNTDTSEKKGFNNSEKDEKARNLISKNYKNYNELDKNVHTLVNSIISLLEEGNGSD
MSP3.8 D7     NKSLEEKQIDKNTDTSEKKGFNNSEKDEKARNLISKNYKNYNELDKNVHTLVNSIISLLEEGNGSD
MSP3.8 D9     NKSLEEKQIDKNTDTSEKKGFNNSEKDEKARNLISKNYKNYNELDKNVHTLVNSIISLLEEGNGSD
MSP3.8 D10    NKSLEEKQIDKNTDTSEKKGFNNSEKDEKARNLISKNYKNYNELDKNVHTLVNSIISLLEEGNGSD
MSP3.8 D11    NKSLEEKQIDKNTDTSEKKGFNNSEKDEKARNLISKNYKNYNELDKNVHTLVNSIISLLEEGNGSD
MSP3.8 D12    NESLEEKQIDKNTDTSEKKGFNNSENDEKARNLISKNYKNYNELDKNVHTLVNSIISLLEEG
MSP3.8 D13    NKSLEEKQIDKNTDTSEKKGFNNSEKDEKARNLISKNYKNYNELDKNVHTLVNSIISLLEEGNGSD
MSP3.8 T1     NKSLEEKQIDKNTDTSEKKGFNNSEKDEKARNLISKNYKNYNELDKNVHTLVNSIISLLEEGNGSD
MSP3.8 T2     NKSLEEKQIDKNTDTSEKKGFNNSEKDEKARNLISKNYKNYNELDKNVHTLVN
MSP3.8 T3     NKSLEEKQIDKNTDTSEKKGFNNSEKDEKARNLISKNYKNYNELDKNVHTLVNSIISLLEEGNGSD
MSP3.8 T4     NKSLEEKQIDKNTDTSEKKGFNNSEKDEKARNLISKNYKNYNELDKNVHTLVNSIISLLEEGNGSD
MSP3.8 T5     NKSLEEKQIDKNTDTSEKKGFNNSEKDEKARNLISKNYKNYNELDKNVHTLVNSIISLLEEGNGSD
```
